# Relaxation Dynamics in Dihydroxychalcones: Insights from Ultrafast Spectroscopy and Quantum Computations

**DOI:** 10.1021/acsphyschemau.5c00057

**Published:** 2025-09-19

**Authors:** Simin Roshan, Michael Hymas, Matthieu M. Mention, Florent Allais, Vasilios G. Stavros, Reza Omidyan

**Affiliations:** † Department of Chemistry, 48437 University of Isfahan, Isfahan 81746-73441, Iran; ‡ School of Chemistry, 2707 University of Birmingham, Edgbaston B15 2TT, United Kingdom; § URD Agro-Biotechnologies Industrielles (ABI), CEBB, AgroParisTech, Pomacle 51110, France

**Keywords:** ultrafast spectroscopy, UV protection, chalcones, conical intersections, photostability, ultrafast relaxation

## Abstract

Chalcones present a potentially promising form of natural photoprotection for inclusion in sunscreen formulations. Here, using femtosecond transient electronic absorption spectroscopy and high-level quantum computations, we explore the differing photophysics of two members of the chalcone family: 4,4’-dihydroxychalcone and 4,4’-dihydroxychalcone-α-methoxylate. From experiment, trapped excited-state population in 4,4’-dihydroxychalcone is alleviated by functionalization at the α carbon, affording vast acceleration in nonradiative deactivation. From theory, the ultrashort excited-state lifetime of the α-substituted analog is explained by a barrierless S_1_/S_0_ conical intersection, providing a route for ultrafast internal conversion, whereas a significant potential energy barrier prohibits the excited system from approaching this conical intersection in the nonsubstituted chalcone. These observations are supported by results from nonadiabatic dynamics simulations. Our investigations elucidate how targeted chemical modifications can perturb potential energy surfaces, resulting in distinct photophysical behaviors. We demonstrate that chalcones’ deactivation mechanisms are sensitive to substitution at the aliphatic bridge connecting the two aromatic rings.

## Graphical abstract

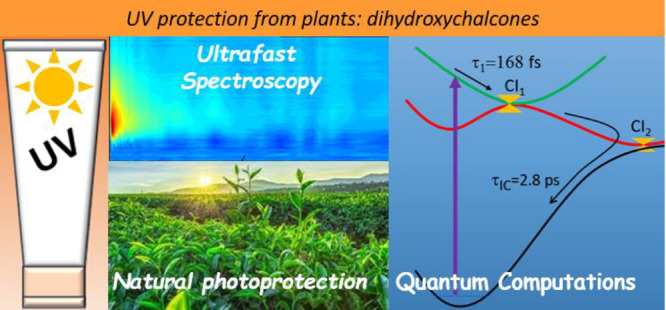

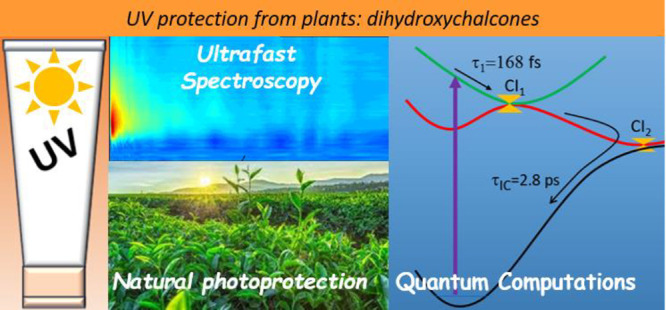

## 1 Introduction

Despite the beneficial role of solar radiation in life, it is well known that overexposure to ultraviolet (UV) radiation can damage skin, cause DNA mutations, and lead to skin cancer.^1,2^ While a negligible amount of UVC radiation reaches the Earth, the amount of terrestrial UVA and UVB radiation (respectively, 315–400 nm and 280–315 nm) is substantial. Sunscreen formulations are designed to protect the skin against the harmful effects of UV radiation by absorbing or scattering harmful rays.^3,4^ Upon absorbing UV radiation, molecules typically undergo a series of nonradiative relaxation processes to release the energy and, ideally, recover their ground state to complete the photochemical cycle, thereby enabling sustained photoprotection.^5,6^ In sunscreen systems, correlating the molecular structure and function requires detailed insight into the relaxation pathways available following photoexcitation. A major issue is that popular UVA absorbers, like avobenzone, are not photostable.^7^ Further optimization is therefore required in order to develop compounds that exhibit model sunscreen characteristics, including, but not limited to, greater UVA/UVB absorption, minimal photoproduct formation, maximal photostability, higher antioxidant activity, and low risk of photosensitization or allergy.^8,9^

Inspiration to develop a new generation of UV filters complying with these characteristics comes from nature. A group of natural UV absorbers are the bioactive chalcones, or (*E*)-1,3-diphenyl-2-propen-1-ones,^10^ which are promising sunscreen agents due to their high UV absorption in the UVB and UVA regions.^11–16^ Chalcones are flavonoid-type phenolic phytochemicals that serve as critical precursors in the flavonoid biosynthesis pathway.^17–19^ These versatile compounds are found in a vast catalog of consumable sources, such as vegetables and fruits, including apples, tomatoes, licorice, teas, and spices.^20–22^ Structurally, chalcones are characterized by two aromatic rings linked by an α, β-unsaturated carbonyl bridge, which confers distinct photochemical and biosynthetic properties to the species.^19,23,24^ Several patents are available for chalcones and their derivatives for biological activities as anticancer, anti-inflammatory, antimicrobial, antifungal, and neuroprotective agents.^24–28^ The high antioxidant activity and low toxicity of chalcones are crucial to their application on skin.^15,19,21,29^ It is possible that sunscreen agents based on chalcone derivatives can be developed for large-scale commercial production, as these systems are readily synthesized and functionalized to form derivatives that have a broad range of absorption maxima (λ_max_) (∼280–365 nm) and relatively high molar absorptivity (∼20–30,000 M^–1^cm^–1^).^15,20,30,31^

In the present work, we investigate the photochemistry and photophysics of two purposely designed chalcone derivatives in ethanol, namely 4,4’-dihydroxychalcone (**A**) and 4,4’-dihydroxychalcone-α-methoxylate (**B**) (see Figure 1). We aim to address the differing photochemistry of these systems despite their shared molecular scaffold.

**Figure 1:**
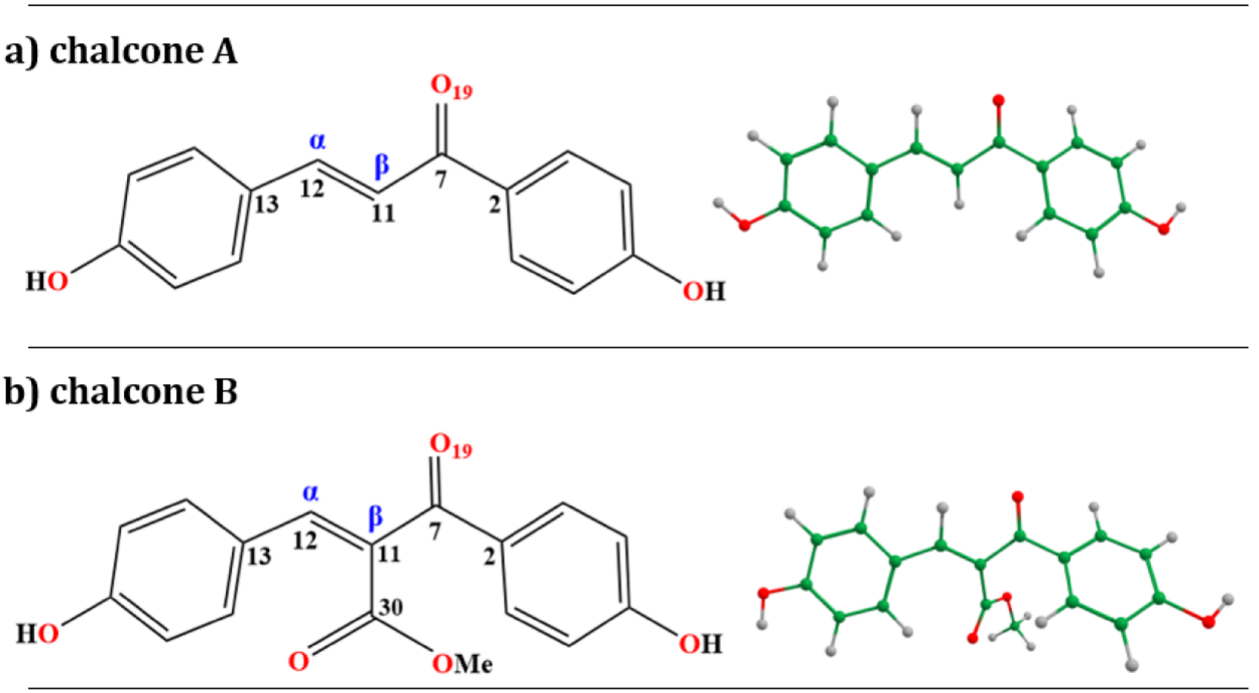
Structural formula (left) and optimized geometry (right) of the most stable isomers of chalcone **A** and chalcone **B**, determined at the RI-MP2 theoretical level.

## 2 Methods

### 2.1 Computational Methods

The conformational landscapes of **A** and **B** were determined based on a novel metadynamics (MTD) scheme for conformational ensembles and stable geometry identification, using the Conformer-Rotamer Ensemble Sampling Tool (Crest V. 2.12).^32,33^ For **A** and **B**, 30 and 66 geometries were identified, respectively. All conformers underwent geometry optimization using density functional theory (DFT) at the B3LYP/cc-pVDZ level, incorporating the D3 dispersion correction factor. Of the resulting conformers, the 10 lowest-energy structures of **A** and 60 of **B** were subjected to a final optimization using the RI-MP2 and DFT (ωB97XD and PBE0 functionals) theoretical levels using the cc-pVDZ^34^ basis set. The 10 lowest-lying structures are presented in Table S1. As shown, structure “**1**” (**A** and **B**, illustrated in Figure 1) has been assigned as the most stable, and we have selected this system for further calculations.

Using these ground state optimized geometries, the excited states, corresponding electronic transition energies, and respective oscillator strengths of the lowest four singlet excited states have been computed using RI-ADC(2), TD-DFT/ωB97XD,^35^ TD-DFT/PBE0,^36^ and multistate (5 states) complete active space second-order perturbation theory (MS-CASPT2)^37^ using the cc-pVDZ basis set. The active space used in all calculations included 12 electrons in 8 molecular orbitals (6 occupied orbitals and 2 unoccupied orbitals). For the TD-DFT calculations of each system, the PBE0 and ωB97XD functionals were used due to their reliability in similar systems.^38–43^

To investigate the implicit ethanol solvent effects, we have employed the polarizable continuum model^44^ (PCM), implemented in Gaussian 16 ^45^ and OpenMolcas,^46^ as well as the COSMO model^47,48^ implemented in Turbomole (V. 6.6).^49,50^ The TD-DFT calculations were undertaken using the Gaussian 16 program.^45^ The RI-MP2 and RI-ADC(2) calculations were performed using the Turbomole program suite (V. 6.6). All state-averaged complete active space self-consistent field (SA-CASSCF)/MS-CASPT2 ^51,52^ calculations were performed using OpenMolcas (V. 18.09).^46,53^ The minimum energy conical intersection (CI) geometry was determined using a SA-CASSCF (6,6)^54^ model. The active space of (6, 6) was chosen due to its reliability in similar systems^39–41,55^ and its affordable computational cost.

To explore the ground and excited state potential energy surfaces (PESs), linear interpolation in internal coordinates (LIIC), based on the ADC(2) theoretical level, was undertaken. In addition, nonadiabatic dynamics (NAD) simulations were performed, employing the trajectory surface hopping (TSH) method based on the TD-DFT theoretical model, using Newton-X software (V. 2.4)^56,57^ interfaced with the Gaussian program suite. In both systems, dynamics were initialized from the (bright) S_2_ (^1^ππ*) state based on the TD-ωB97XD approach. Our choice of model for determining excited-state deactivation processes has been validated in previous reports.^38–40,42,43^ A set of 500 initial molecular geometries and momenta was created by sampling a Wigner distribution of ground-state harmonic frequencies. At each Wigner-sampled geometry, the vertical excitation energies and oscillator strengths for transitions to the first four singlet states were computed. UV–visible absorption spectra of **A** and **B** were simulated using the nuclear ensemble approach.^58^ Nonadiabatic coupling effects between the S_2_, S_1_, and S_0_ states were mimicked by Tully’s fewest switches surface hopping algorithm^59,60^ with decoherence correction (*a* = 0.1 hartree). In standard TSH, each trajectory evolves independently, and the electronic wave function can remain unrealistically coherent across states, even after a hop. This leads to inaccuracies in simulating nonadiabatic transitions. To mitigate this, Newton-X implements energy-based decoherence corrections, which introduce a damping mechanism to reduce the off-diagonal elements of the electronic density matrix. This correction mimics the natural loss of coherence due to nuclear motion and environmental interactions.^60,61^

### 2.2 Synthesis

Chalcone **A** was synthesized in one step using a Claisen–Schmidt condensation, as described in a previous study,^62^ using ethanol as solvent and HCl as catalyst (see Scheme 1).

**Scheme 1:**
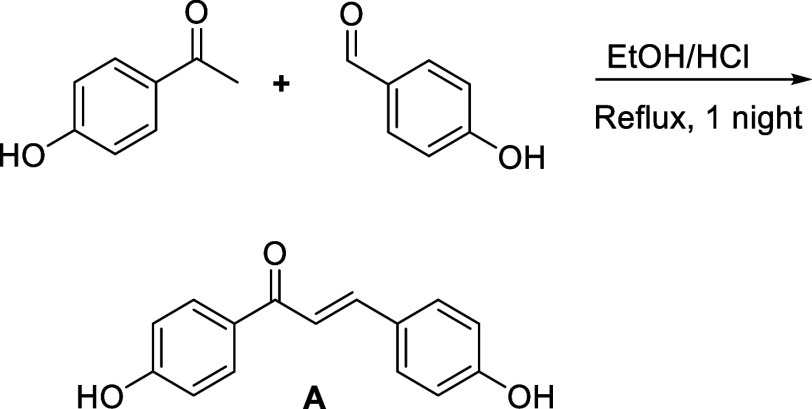
Synthesis (Claisen-Schmidt Condensation) of Chalcone **A**

The synthesis of substituted chalcone **B** was carried out in two steps from 4-hydroxyacetophenone: nucleophilic substitution of the latter with dimethylcarbonate, followed by l-proline-organo-catalyzed Knoevenagel condensation of the resulting β-keto ester intermediate with 4-hydroxybenzaldehyde in ethanol (see Scheme 2).

**Scheme 2:**
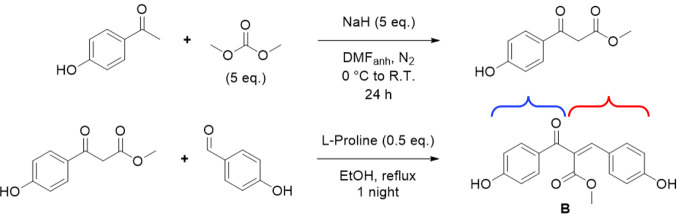
Synthesis (Nucleophilic Substitution (Top) and Knoevenagel Condensation (Bottom)) of Chalcone **B**. The Cinnamoyl (Red) and Benzoyl (Blue) Ends of the Chalcone are Shown by Brackets

In the first step, sodium hydride (1.48 g, 37.0 mmol) was suspended in *n*-hexane (10 mL) under a N_2_ atmosphere and left to settle for 10 min before the solvent was removed. The procedure was repeated a second time before anhydrous DMF (22 mL) was added. The reaction was placed in an ice bath to maintain the temperature at 0 °C. A solution of 4-hydroxyacetophenone (1.00 g, 7.33 mmol) in 10 mL of anhydrous DMF was added dropwise to the NaH suspension over 1.5 h. After completion of the addition, the solution was left stirring for 15 min. Dimethylcarbonate (3.2 mL, 38.0 mmol) was added dropwise over 1 h, and the reaction was left stirring overnight at room temperature. The reaction mixture was cooled in an ice bath, poured into an iced solution of 1 M HCl (50 mL), and extracted with ethyl acetate (4 × 20 mL). The combined ethyl acetate fractions were washed with water (40 mL), a solution of 5% citric acid (2 × 20 mL), a solution of 5% sodium bicarbonate (2 × 20 mL), and then again with water (40 mL), prior to being dried over anhydrous magnesium sulfate (MgSO_4_) and concentrated. The desired β-keto ester intermediate was obtained with no further purification as a yellow solid (1.03 g, 5.30 mmol, 73%).

In the second step, the β-keto ester intermediate (1.00 g, 5.13 mmol), 4-hydroxybenzaldehyde (0.63 g, 5.17 mmol), and l-proline (0.30 g, 2.61 mmol) were mixed in ethanol. The reaction mixture was stirred at reflux overnight. After cooling to room temperature, ethanol was removed, and the crude product was purified by flash chromatography (cyclohexane:ethyl acetate 8:2). Fractions containing the desired product were combined and concentrated under vacuum to give pure substituted chalcone **B** (0.51 g, 1.71 mmol, 33%). Additional information about this part can be found in Figures S1 and S2.

### 2.3 Spectroscopic Methods

For spectroscopic measurements (excluding determining extinction coefficients), solutions of **A** and **B** were made up to ∼0.5 absorption. All solutions were covered with foil during storage to exclude ambient light. Steady-state UV–visible absorption spectra were recorded using a Cary 60 UV/vis spectrophotometer in either a 1 cm or 1 mm quartz cuvette. Emission quantum yields were recorded using an Edinburgh Instruments FS5 spectrofluorometer with an SC-30 integrating sphere.

Femtosecond transient electronic absorption spectroscopy (fs-TEAS) experiments are outlined in greater detail elsewhere,^63–64^ so details specific to these experiments are presented herein. The “pump” excitation wavelength was set to the centroid of the longest wavelength Gaussian used to fit the UV–visible absorption spectrum of each solution sample (Figure 2). The pump pulse energy was ∼500 nJ, and the beam spot size was ∼350 μm at the sample. The “probe” pulse was a white light supercontinuum that spanned ∼320–720 nm. Pump–probe time delay, Δ*t*, was controlled by a motorized delay stage (<2.5 ns). A 1 mm quartz cuvette, containing the (∼0.5 absorption) sample solution, was translated in the plane perpendicular to the pump–probe geometry (to minimize generated photoproducts being probed by subsequent pulse pairs).

**Figure 2:**
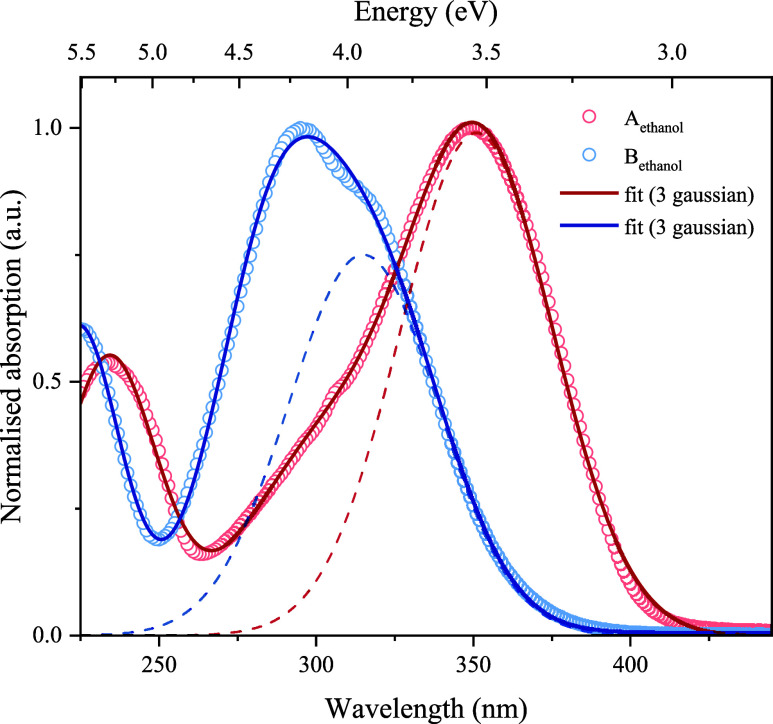
Gaussian functions simulating the absorption spectrum of **A** and **B** in ethanol. Open circles: recorded absorption data. Overlaid: fit (solid) and longest-wavelength Gaussian component (dashed).

To obtain dynamical information from fs-TEAS results, the collected transient absorption spectra were fitted with a sequential global fitting model, implemented in the software package Glotaran.^65^ Evolution-associated difference spectra (EADS), associated with lifetimes extracted and residuals from fitting, are presented in Figures S8 and S9. An instrument response function (IRF) for each pump–probe experiment was evaluated by obtaining kinetic transient absorption traces from solvent-only scans (Figure S10). Heatmaps presented in this manuscript were treated using the KOALA^66^ software to model (and correct) dispersion.

## 3 Results and Discussion

### 3.1 Ground State Geometries

Chalcones, such as **A** and **B**, contain two aromatic rings joined by a three-carbon α, β-unsaturated carbonyl system (Figure 1), and can be found in nature as either their *E* or *Z* isomers.^67^ The optimized structures of the *Z* and *E* isomers of **A** and **B** in the S_0_ state, as well as their relative energies (determined at the MP2/cc-pVDZ theoretical level), are shown in Figure S7 and Table S1. It was found that the *E* isomer of **A** and **B** is more stable than the *Z* isomer (by 0.19 and 0.22 eV, respectively), in agreement with previous studies.^67,68^ The next 9 lowest-lying conformers of **A** and **B** are shown in Table S1. Further details on the conformational search, ground-state optimized geometries, and coordinates can also be found in the Supporting Information.

### 3.2 Experimental and Simulated UV–Visible Properties

Initially, UV-visible absorption spectra of the two chalcones in ethanol (and in 1,4-dioxane to check for solvatochromism, see Figure S3) solutions were recorded (Figure 2). As is characteristic of chalcones, two distinct electronic absorption bands (I and II) can be seen at ∼350 and ∼250 nm, which shift minimally between polar and nonpolar solvents, arising predominantly from transitions located on the cinnamoyl and benzoyl moieties, respectively.^10^ Three Gaussian lineshapes were fit to model these spectra, stimulated by the lowest three bright transitions identified from theory. The centroid of the longest wavelength (350 nm for **A** and 315 nm for **B**, respectively) was selected as the photoexcitation energy in transient absorption experiments (see *infra*). Extinction coefficients were recorded for **A** (ε = 29800 ± 260 M^–1^cm^–1^) and **B** (ε = 26000 ± 120 M^–1^cm^–1^) in ethanol at 350 and 315 nm, respectively (see Figure S4).

Stability under a simulated solar spectrum (Figure S5) was assessed by recording UV–visible absorption spectra following irradiation for 2 h. As is common in photoprotection literature,^69^ an area under curve index (AUCI) between 280 and 400 nm for **A** and **B** was calculated to assess their photostability. This yielded 41% for **A** in ethanol (55% in 1,4-dioxane) and 97% for **B** in ethanol (99% in 1,4-dioxane), classifying **A** as photounstable and **B** as photostable (see Figure S6). The lack of an isosbestic point in absorption spectra throughout irradiation implies photochemistry beyond a simple unimolecular reaction, as is expected from prolonged exposure to a “solar” (i.e., broadband) spectrum.

Vertical transition energies and oscillator strengths have been calculated for the ground-state optimized structures of **A** and **B** (Figure 1) up to the fourth singlet excited state (S_1_–S_4_), both *in vacuo* and in an implicit ethanol solvent (Table 1). We employed different levels of theory: TD-DFT/ωB97XD and PBE0, RI-ADC (2), and MS-CASPT2 (12,8). Valence molecular orbitals (MOs) contributing the most to electronic transitions for these two systems are shown in Figure 3 (further details can be found in Tables S2 and S3).

**Figure 3:**
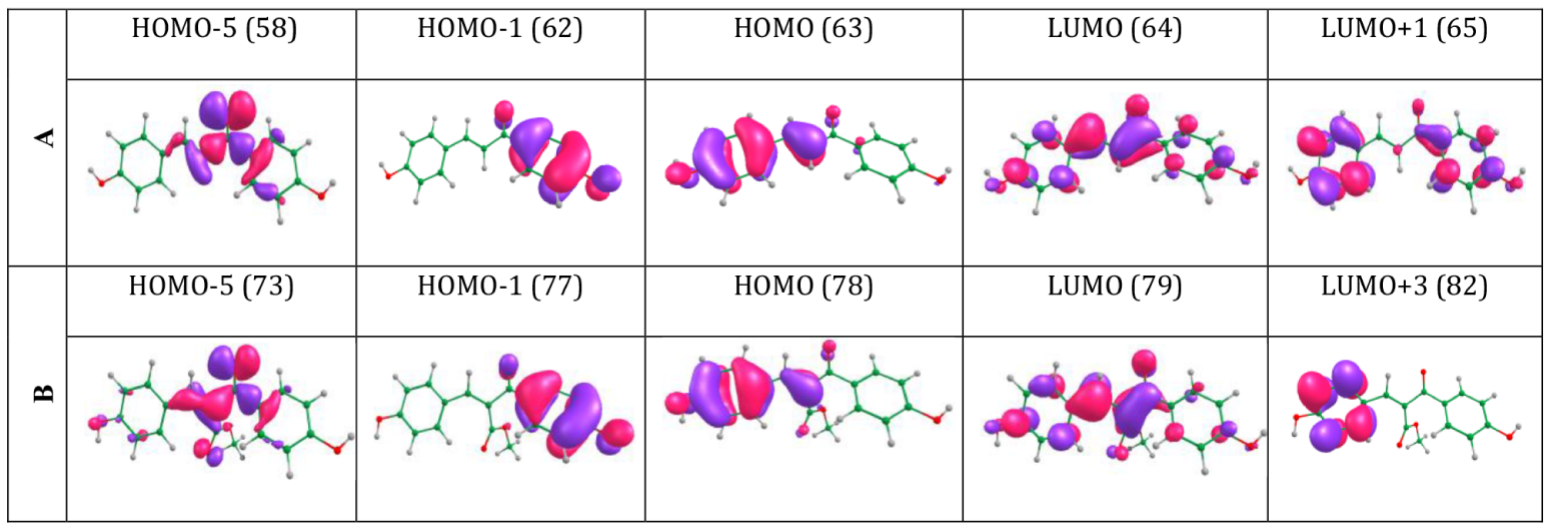
Selected valence molecular orbitals involved in the lowest-lying electronic transitions for **A** and **B**.

**Table 1:** Calculated Vertical Transition Energies and Oscillator Strengths (In Parentheses) for the Lowest Singlet Excited States of **A** and **B** in the Gas Phase and in Implicit Ethanol Solvent^a^

Transition energy/eV					
Excited state	**ADC(2)**		**TD-ωB97XD**	**TD-PBE0**	**MS-CASPT2**
A					
Electronic state	Gas Phase	COSMO/Ethanol	PCM/Ethanol	PCM/Ethanol	PCM/Ethanol
**S**_**1**_ **(nπ** ^*^ **)**	3.31 (0.0003)	3.37 (0.0012)	3.70 (0.0000)	3.48 (0.0000)	3.62 (0.0003)
**S**_**2**_ **(ππ** ^*^ **)**	4.23 (1.000)	3.87 (0.9894)	3.92 (0.9801)	3.55 (0.9623)	3.75 (0.9461)
**S**_**3**_ **(ππ** ^*^ **)**	4.67 (0.0869)	4.39 (0.2211)	4.70 (0.1536)	4.09 (0.0640)	4.61 (0.1676)
**S**_**4**_ **(ππ** ^*^ **)**	4.74 (0.0911)	4.64 (0.0059)	4.85 (0.0041)	4.54 (0.0001)	4.82 (0.0685)
**Gaussian** λ_max (ethanol)_	3.54/350 nm				
**B**					
**S**_**1**_ **(nπ** ^*^)	3.49 (0.0133)	3.56 (0.0535)	3.83 (0.2503)	3.55 (0.3323)	3.88 (0.0082)
**S**_**2**_ (ππ^*^)	4.20 (0.8123)	3.98 (0.8638)	4.01 (0.8827)	3.74 (0.6028)	4.03 (0.8393)
**S**_**3**_ **(ππ** ^*^)	4.67 (0.0154)	4.41 (0.2048)	4.73 (0.1447)	4.03 (0.0354)	4.82 (0.1149)
**S**_**4**_ **(ππ** ^*^)	4.79 (0.1617)	4.65 (0.0015)	4.85 (0.0012)	4.40 (0.0524)	5.25 (0.0805)
**Gaussian** λ_max (ethanol)_	3.94/315 nm				

a The multistate CASPT2 results have been determined based on CASSCF with an active space of 12 electrons over 8 orbitals

According to ADC(2) results, the first electronic transition (S_1_←S_0_) for both systems originates from the fifth orbital below the highest occupied molecular orbital to the lowest unoccupied molecular orbital (LUMO←HOMO–5). This transition has ^1^nπ* character and is, therefore, expectedly optically dark, owing to low oscillator strength (see Table 1). The second electronic transition in both systems, S_2_←S_0_, has been assigned to a (bright) ^1^ππ* state arising from a LUMO←HOMO transition. The main orbital contributions in the third electronic transition for **A** and **B** are LUMO+1←HOMO or LUMO+3←HOMO, respectively, assigned as a ^1^ππ* state in each case. The lowest-lying electronic transition at the MS-CASPT2/cc-pVDZ level and from TD-ωB97XD and TD-PBE0 models were also calculated (see Tables S2 and S3). From consideration of all theoretical levels, the first ^1^ππ* state (S_2_ state) is primarily responsible for UV absorption in both systems; we have therefore focused mainly on this state and neglected higher-energy electronic transitions. As shown in Table 1, for **A**, this transition is within 3.55–3.92 eV in implicit ethanol at the ADC(2), TD-ωB97XD, TD-PBE0, and MS-CASPT2 levels of theory. Similarly, for **B** in implicit ethanol, this transition has been determined to be within 3.74–4.03 eV.

The small discrepancies between levels of theory and the consistency between different methods demonstrate the reliability of the selected theoretical models in describing the photochemistry of these chalcones. All selected models reasonably approximate the Gaussian peaks used to simulate experimental UV absorption profiles of ethanolic **A** and **B** (3.54 and 3.94 eV correspond to 350 and 315 nm, respectively).

### 3.3 Dynamical Properties

To inspect the dynamical properties of both chalcones in solution, femtosecond transient electronic absorption spectroscopy (fs-TEAS) was performed, with photoexcitation at the centroid of the longest wavelength Gaussian identified from UV–visible absorption spectra in ethanol (Figure 4).

**Figure 4:**
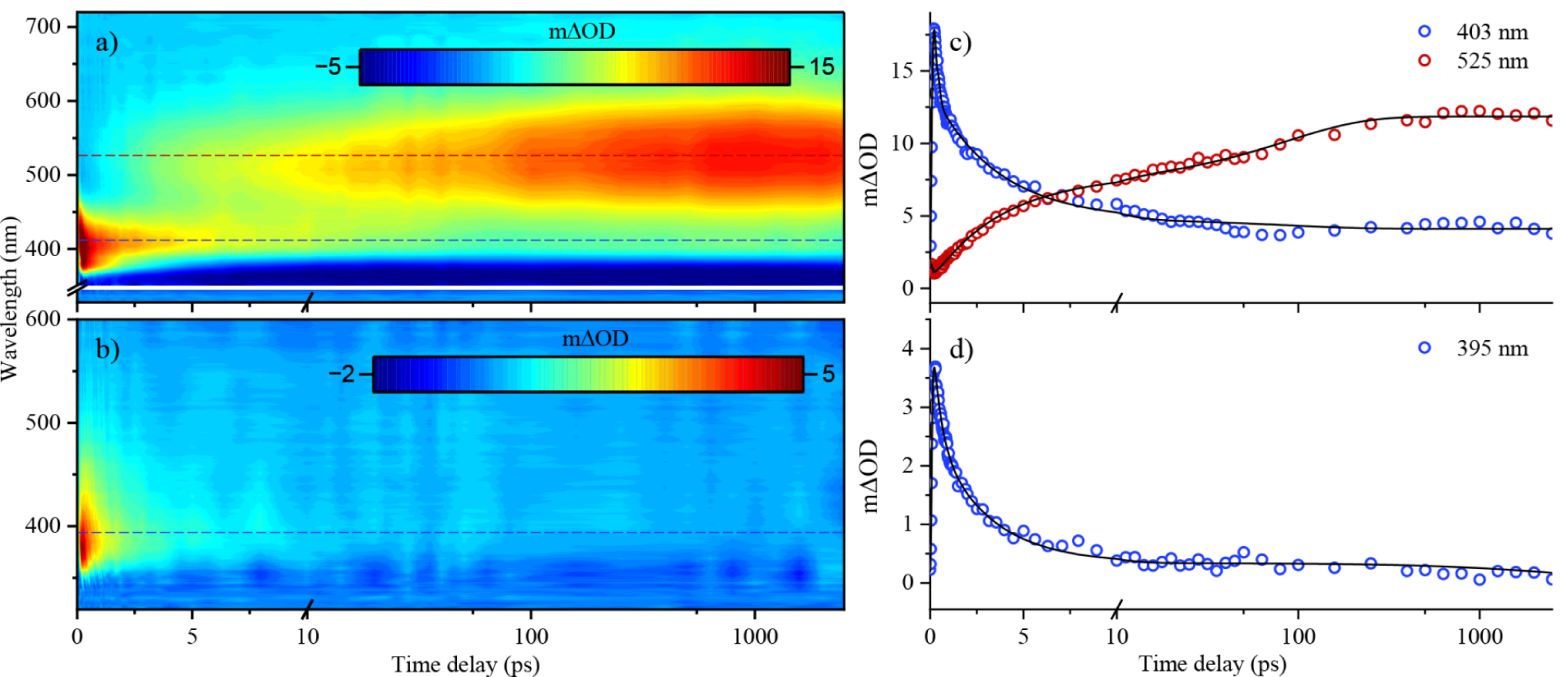
Collected transient absorption spectra and kinetic transient absorption traces at probe wavelengths labeled (with fits from global sequential analysis overlaid) of **A** (a, c) and **B** (b, d) in ethanol, photoexcited at 350 and 315 nm, respectively. Time scales are linear up to 10 ps and logarithmic thereafter.

Collected fs-TEAS data were analyzed by a global sequential fitting scheme implemented in the Glotaran software.^65^ Several lifetimes (see Table 2), with the corresponding EADS (see Figure S8), were extracted. Residuals from fits are also presented in Figure S9. To emphasize, although the same number of lifetimes were extracted from **A** and **B**, this does not imply that both species are undergoing equivalent photophysical processes.

**Table 2:** Lifetimes are Extracted from Global Sequential Fitting of Collected Transient Absorption Data.^a^

	**A**_ethanol_	**B**_ethanol_
τ_1_ (ps)	0.298 ± 0.058	0.168 ± 0.060
τ_2_ (ps)	3.516 ± 0.058	0.408 ± 0.060
τ_3_ (ps)	85.527 ± 0.665	2.885 ± 0.060
τ_4_ (ps)	>2500	>2500

a Half of IRF (Figure S10) is reported as the error in lifetime, except in cases where the error extracted from fitting is Greater

Broadly, τ_1_ and τ_2_ in **A**, capturing concurrent excited state absorption (ESA) at ∼400 nm and ground state bleach (GSB) at ∼350 nm, are together assigned to evolution of the excited state population across the S_2_ PES. Subsequently, τ_3_ reflects diminishing ESA at ∼400 nm before a long-lived signature at ∼520 nm persists beyond our experimental time window (τ_4_). From this, and unrecovered GSB, we infer that electronic population in **A** remains either trapped in an excited state (possibly in a nearby triplet level, based on the large, red-shifted ESA feature and similar findings from literature),^70^ or facilitates excited state photochemistry.

In **B**, however, all excited-state processes appear complete within a few ps; τ_1_ and τ_2_ collectively capture a decaying and red-shifting ESA signal, arising from projection of the generated S_2_ population into a subsequent excited state, and τ_3_ reflects depopulation of S_2_ into the electronic ground state, subsequently forming the photoproduct (lasting beyond our experimental time window). τ_4_ therefore captures a convolution of unrecovered GSB and the photoproduct absorption profile.

Qualitatively similar dynamical features were seen in fs-TEAS undertaken in 1,4-dioxane (see Figure S11 and Table S4), indicating broadly solvent-independent photophysics for the chalcones. It should be noted that only three lifetimes were extracted from **B** in 1,4-dioxane; τ_1_ therefore likely captures a convolution of processes in the initially photoexcited state (as τ_1_ and τ_2_ did together in ethanol).

### 3.4 Nonadiabatic Dynamics (NAD) Simulations

To investigate the time evolution of **A** and **B** and compare these findings to ultrafast experiments, we undertook nonadiabatic dynamics (NAD) simulations using the trajectory surface. The decoherence-corrected fewest switches surface hopping (DC-FSSH)^59,60^ algorithm was employed to take into account nonadiabatic coupling contributions between the S_2_, S_1_ and S_0_ levels. Accordingly, the UV–visible absorption spectra of both systems were simulated using the nuclear ensemble approach^58^ for the S_2_ state and show absorption maxima at 3.48 eV (356 nm) and 3.92 eV (316 nm) for **A** and **B**, respectively, in good agreement with Gaussian fits to experimental spectra (350 and 315 nm for **A** and **B,** respectively).

The spectral window for dynamics was, therefore, selected as 3.48 ± 0.10 eV for **A** and 3.92 ± 0.10 eV for **B** (indicated by shaded areas in Figure S12).

A total of 97 and 94 trajectories were computed for **A** and **B,** respectively, with dynamics for both systems simulated for a maximum of 1500 fs. The velocity Verlet algorithm^71^ was used to integrate classical equations of motion with a time step of 0.5 fs. The trajectory surface hopping (TSH) method was initialized from the (bright) second excited state, S_2_ (^1^ππ*), based at the TD-ωB97XD theoretical level.

Figure 5 shows the time evolution of ground and excited state energies for selected trajectories of **A** and **B**. In both systems, the S_2_ state population is completely transferred to S_1_ within the initial ∼150 fs. However, within 1500 fs, no subsequent depopulation of S_1_ occurs in **A**. The S_1_ excited state lifetime in **A** should therefore be longer than 1500 fs but cannot be evaluated computationally with any confidence. By contrast, for **B**, 90% of the trajectories exhibit relaxation from S_1_ to S_0_, *via* an S_1_/S_0_ CI, within ∼650 fs (see Figure S13; 597 fs for the selected trajectory in Figure 5).

**Figure 5:**
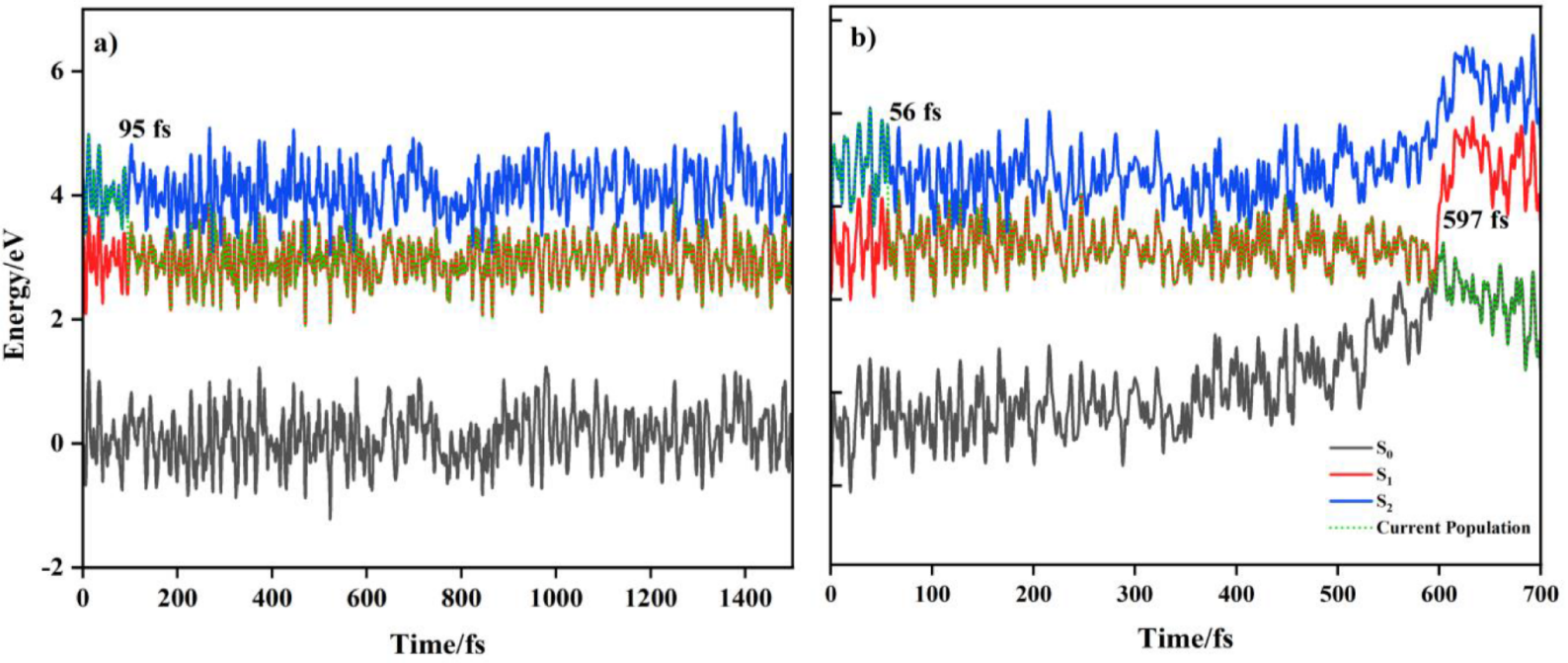
Energy profiles of selected trajectories for (a) **A** and (b) **B**. The gray, red, and blue curves represent the S_0_, S_1_ and S_2_ electronic states, respectively. In addition, the green dotted line indicates the current state population at each time step. The times at which population transfers from one state to another are labeled (i.e., from S_2_ → S_1_ in **A** and from S_2_ → S_1_ and S_1_ → S_0_ in **B**).

Analysis of the evolution of **B**’s structure in trajectories allowed identification of nuclear motions facilitating nonadiabatic coupling (i.e., characterizing the CI). As anticipated, dynamical simulations of **B** revealed that a twist around the α, β-double bond (i.e., *E*-*Z* isomerization, as is well-documented for chalcones)^11,13,72^ is the main coordinate for S_1_ relaxation to S_0_. For all trajectories, the CI is facilitated by a twist around the double bond until the C_13_–C_12_–C_11_–C_7_ dihedral angle changes from 0° to 90° (see Figure 6a).

**Figure 6:**
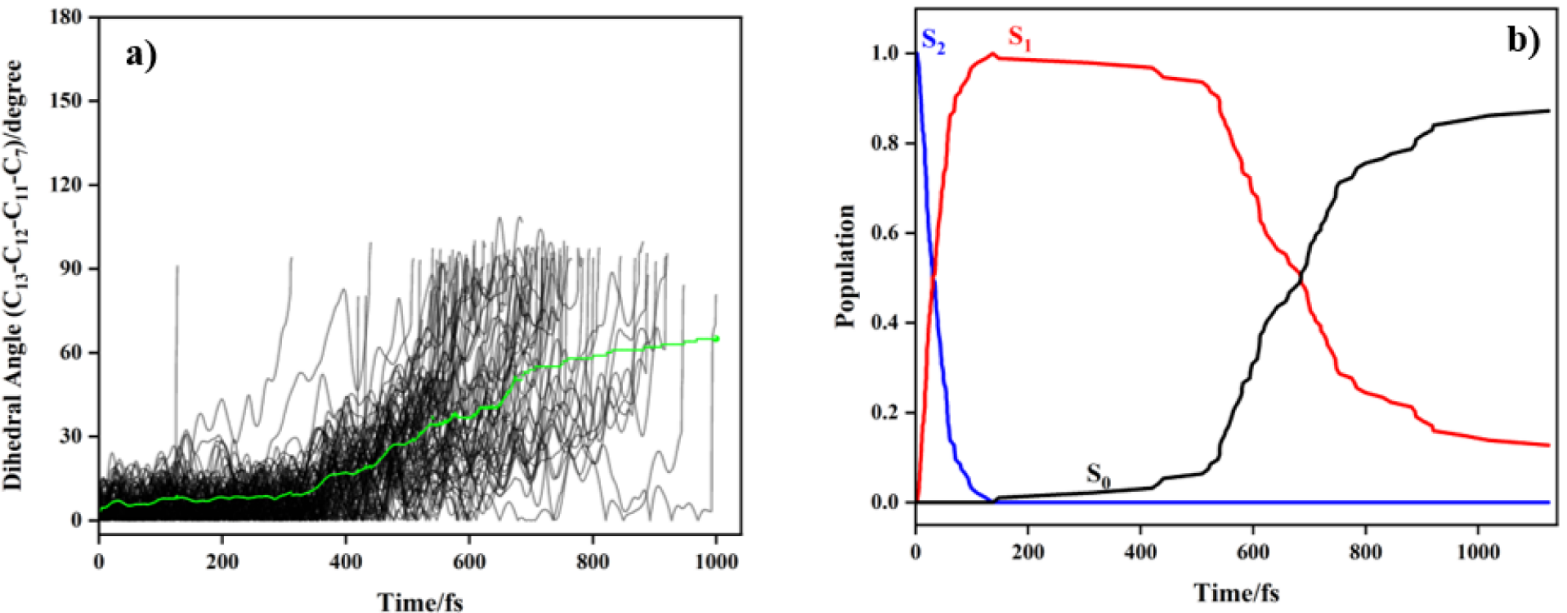
(a) Time evolution of the dihedral angle of (C_13_–C_12_–C_11_–C_7_, or α, β-double bond dihedral) of **B** against simulation time, presented for 94 trajectories. In addition, the green line indicates the average dihedral angle versus simulation time from the trajectories. (b) Population of the ground (black), S_1_ (red), and S_2_ (blue) states against simulation time (averaged over 94 trajectories) for **B**.

To determine the S_2_ and S_1_ excited state lifetimes for **B**, we analyzed the time evolution of the nonadiabatic population in the two excited states (Figure 6b). The time for population transfer to occur from S_2_ to S_1_ is taken as indicative of the time taken to reach an S_2_/S_1_ CI. The same approach was applied for determining the time taken to reach the S_1_/S_0_ CI. S_2_ state population was transferred to S_1_ within ∼140 fs, and the population subsequently decays to S_0_. From fitting the state population with a sigmoidal function^38^ (see Figure S13 and eq S2), the lifetime of the S_2_ excited state was extracted as 36 ± 0.7 fs, and the lifetime of the S_1_ excited state as 647 ± 3.0 fs. As a result, the total internal conversion (IC) lifetime for **B** is τ_IC_ = 36 + 647 fs, in reasonable accord with the 2.89 ps lifetime extracted from the experiment. Most of the excited population in **A** is predicted to become trapped in the S_1_ excited state, and either deactivates *via* luminescence (although an emission quantum yield (QY) of ∼1% was observed) or leads to excited state chemistry, explaining the observed photodegradation.

### 3.5 Conical Intersections (CIs) and Potential Energy Surfaces (PESs)

To further explore the excited-state relaxation mechanisms of **A** and **B**, we searched for accessible CIs in both chalcones. As discussed *supra*, our nonadiabatic dynamics simulations of **B** revealed that rotation around the α, β-double bond directs relaxation of the excited system from S_2_ to S_1_ and then from S_1_ to S_0_. Initializing from a slightly twisted structure (Figure 1), we optimized the S_2_/S_1_ and S_1_/S_0_ CIs for both **A** and **B** with the SA-CASSCF (6,6)/cc-pVDZ theoretical model. These optimized CI geometries are presented in Figure 7.

**Figure 7:**
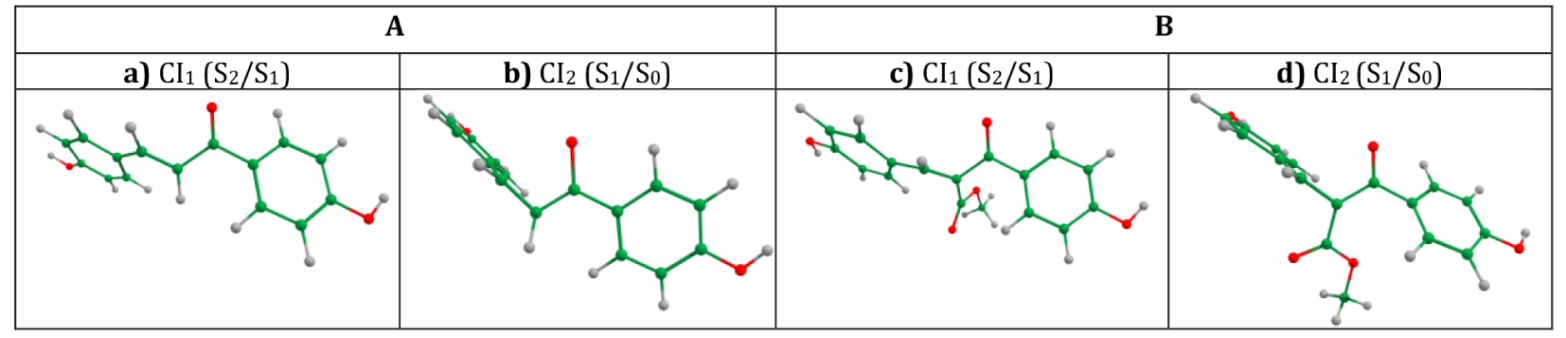
Optimized geometries of **A** (a, b) and **B** (c, d) at CIs, determined at the SA-CASSCF (6,6)/cc-pVDZ theoretical level.

Inspection of both optimized CIs in **A** and **B** reveals twisting about the α, β-double bond with respect to the equilibrium S_0_ geometry. This deformation is more pronounced in CI_2_ (S_1_/S_0_). The C_13_–C_12_-C_11_–C_7_ dihedral angle, responsible for this twisting coordinate, differs by ∼50° in CI_1_ (S_2_/S_1_) compared to both systems’ Franck–Condon (FC) geometry, and by ∼90° in CI_2_, (S_1_/S_0_), corresponding to a perpendicular arrangement of the two benzene rings in each system. The Cartesian coordinates of the optimized CI structures can be found in the Supporting Information.

Locating possible CIs does not guarantee a related nonradiative deactivation process. We therefore calculated points along PESs for the ground and relevant excited electronic states (S_1_^1^nπ*, and S_2_^1^ππ*) along a LIIC, based at the RI-ADC(2)/cc-pVDZ theoretical level. The LIIC potential energy cuts connect the FC geometry to the optimized CIs (CI_1_ and CI_2_). These interpolated PESs are listed in Figure 8. As mentioned above, excitation to S_2_ (^1^ππ*), with significant oscillator strength, is responsible for UV absorption in both chalcones and therefore governs their respective photophysics.

**Figure 8:**
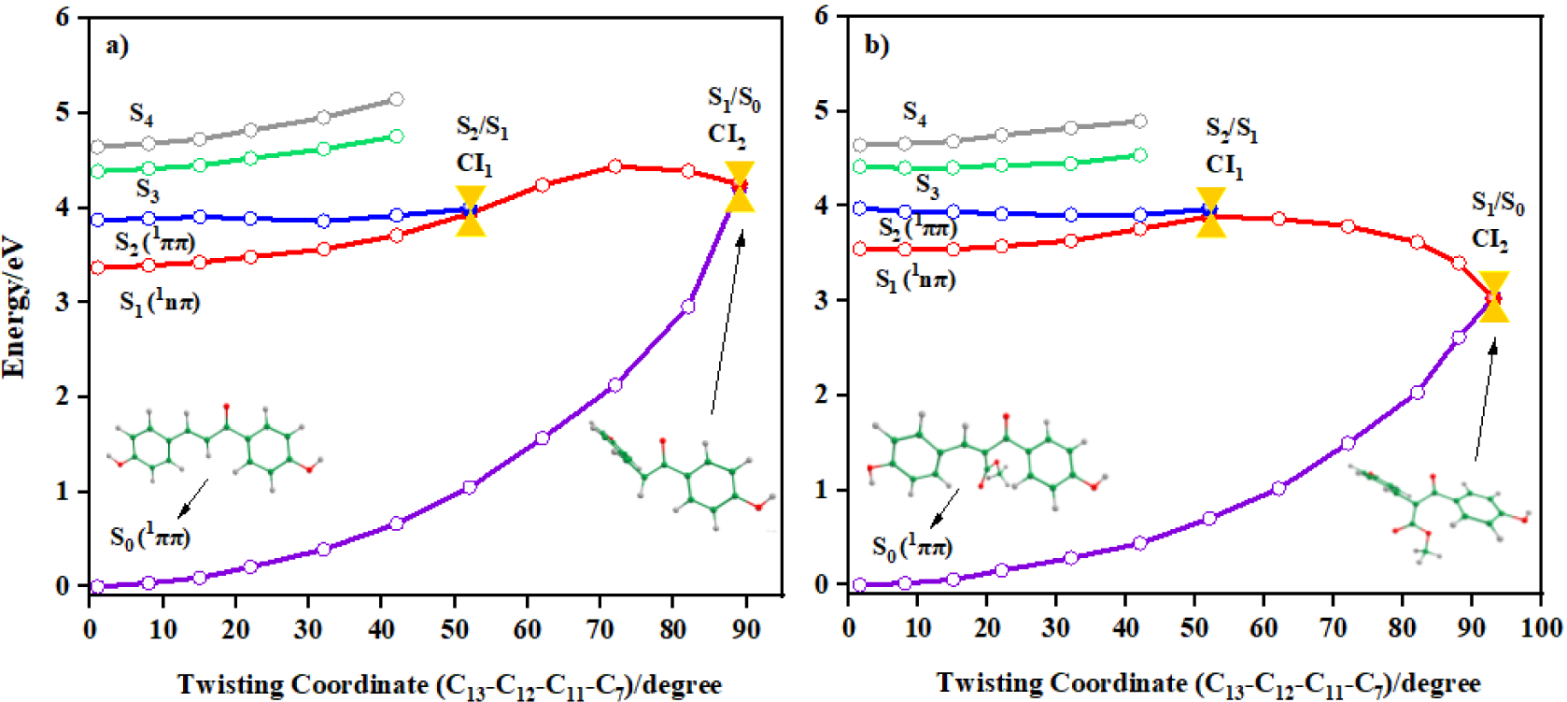
Potential energy surfaces of the ground and excited states of (a) **A** and (b) **B**, calculated at the ADC(2)/cc-pVDZ level of theory, along LIIC (in implicit ethanol solvent).

As the S_2_ PES plateaus in both molecules (i.e., is barrierless), excited state population is expected to proceed unhindered toward CI_1_. Hereafter, PES topologies differ between those of **A** and **B**. In **A**, the S_1_ PES increases markedly in energy, furnishing a large (∼0.90 eV) barrier and thereby hindering the excited-state population from accessing CI_2_ (S_1_/S_0_ crossing). Population trapping in S_1_ is therefore likely in **A**. This is reflected in both experiment and theory (*cf*. Figures 4 and 5). For **B**, following CI_1_ the S_1_ PES decreases in energy until CI_2_. This CI (S_1_/S_0_) has also been predicted to be significantly lower (∼0.55 eV) in energy compared to the vertical S_2_ (^1^ππ*) energy. This crossing, therefore, appears to be accessible to the excited-state population, leading to deactivation *via* IC.

The contribution of excited triplet states (mainly T_1_) to the deactivation of **A** and **B** was also investigated. We determined the PES profile of T_1_ for both systems along the LIIC coordinate; the results are shown in Figure S14. As shown, intersystem crossing (ISC) is unlikely to contribute significantly to the relaxation mechanisms of either system; the energies of triplet state (T_1_) have been predicted to be more than 0.52 eV away from the vertical S_1_ energy. Also, the S_1_/T_1_ curve crossings have been predicted either not to occur (as in **B**) or happen at the end of the reaction coordinate (as in **A**), following a large barrier. This barrier could be quite effective in hindering the wavepacket from approaching this ISC point in **A**. Furthermore, the spin–orbit coupling (SOC) between S_1_ and T_1_ is predicted to be negligible; according to El-Sayed’s rule,^73^ SOC is most efficient between states of different symmetry and electronic configuration. Both S_1_ and T_1_ exhibit the same ππ* character in **A** and **B**, so SOC (and therefore ISC) is expected to be minimal.

Despite the shared scaffold of chalcones **A** and **B**, their distinct photophysical behaviors warrant further examination. The additional α-methoxylate substitution on the C=C bond of aliphatic bridge in chalcone **B** clearly plays a crucial role in governing its photophysics. To explore this, we analyzed the electronic structures (i.e., MOs) involved in the first ^1^ππ* state of both systems. As previously established, this ^1^ππ* state predominantly arises from a LUMO←HOMO single-electronic transition. Evolution of the relevant MOs along the reaction coordinate is depicted in Figure S15. Additionally, variations in the HOMO–LUMO energy gap along the same reaction coordinate for both systems are presented in Figure S16.

We have analyzed the HOMO and LUMO of **B** in relation to the contribution of the additional α-methoxylate, to better understand this group’s photophysical role. A natural bond orbital (NBO) analysis,^74,75^ conducted using Gaussian 16 and Chemissian^76^ programs, was undertaken. The percentage contributions of α-methoxylate to the HOMO and LUMO of **B** are presented in Figure S15. As illustrated, at the initial stages of the reaction coordinate, the contribution of α-methoxylate to these orbitals is minor (7–15%), resulting in the S_2_ profile of **B** closely resembling that of **A**, specifically from the FC region to the S_2_/S_1_ conical intersection (CI_1_). However, beyond CI_1_, the group’s contribution to both HOMO and LUMO becomes more significant, increasing to 60% at the S_1_/S_0_ CI. This larger contribution of α-methoxylate in **B**’s electronic structure (i.e., MOs) involved in the S_1_–S_0_ transition significantly decreases the HOMO–LUMO gap (Figure S16) and stabilizes the S_1_/S_0_ CI. This effect consequently enhances the deactivation dynamics in chalcone **B**.

## 4 Conclusions

Femtosecond transient electronic absorption spectroscopy, *ab initio* excited-state calculations, and nonadiabatic dynamics simulations have been employed to reveal the excited-state deactivation mechanisms of dihydroxychalcones **A** and **B**, photoexcited to their S_2_ (^1^ππ*) state. The ultrashort excited state lifetime of chalcone **B** (determined as ∼2.8 ps from experiment and ∼0.70 ps from theory) has been explicated by an accessible S_1_/S_0_ conical intersection in the excited-state potential energy curves without any barrier. Likewise, chalcone **A**’s observed photodegradation has been explained by a large barrier in the S_1_ potential energy surface, precluding efficient S_1_/S_0_ population transfer and thereby leading to excited-state chemistry. It has been predicted that twisting around the C=C double bond (i.e., photoisomerization) is responsible for the nonradiative deactivation in both systems and that this is greatly favored in **B**. Our findings demonstrate that the α-methoxylate substitution directly influences the electronic structure and potential energy surfaces of dihydroxychalcone, particularly in regions where the molecular configuration deviates significantly from the Franck–Condon region. In these regions of configuration space, the additional group alters the potential energy surface gradient substantially and eases approach of the excited wavepacket toward the S_1_/S_0_ conical intersection, impacting relaxation dynamics and shortening the excited-state lifetime.

While further theoretical and experimental studies are necessary to comprehensively explore the photodynamics of these systems, our results indicate that otherwise unpromising chalcones can be readily derivatized to achieve improved photostability. Consequently, this enhances their photoprotective properties against harmful UV radiation. Moreover, as photostability is an essential attribute of next-generation UV filters, our experimental and theoretical results validate the use of similar natural systems in sunscreen formulations.

## Supplementary Material



## Notes

Details of synthesis process and characterization of **A** and **B**, details of the transient absorption experiments, including solvent responses, evolution associated difference spectra and residuals from global fitting, photostability results, and computational details, including a description of methods for obtaining optimized ground state geometry, electronic transition energies, conical intersections, and potential energy surfaces and nonadiabatic dynamics (PDF)
