# Supplementary material for: Relaxation Dynamics in Dihydroxychalcones: Insights from Ultrafast Spectroscopy and Quantum Computations
Source: ACS Phys Chem Au. 2025 Sep 19;5(6):628–38. doi: 10.1021/acsphyschemau.5c00057 (PMC12670299; doi:10.1021/acsphyschemau.5c00057)
Supplement: Supplementary file 1 [file pg5c00057_si_001.pdf]

Supplementary Information:

Relaxation Dynamics in Dihydroxychalcones: Insights from Ultrafast Spectroscopy and Quantum Computations

Simin Roshan[1†], Michael Hymas[2†], Matthieu M. Mention[3], Florent Allais[3], Vasilios G. Stavros[2*], Reza Omidyan[1,2*]

[1]Department of Chemistry, University of Isfahan, 81746-73441, Isfahan, Iran

[2]School of Chemistry, University of Birmingham, Edgbaston, B15 2TT, United Kingdom

[3]URD Agro-Biotechnologies Industrielles (ABI), CEBB, AgroParisTech, Pomacle 51110, France

†These authors have contributed equally.

1-Synthesis

4-Hydroxybenzaldehyde, 4-hydroxyacetophenone, L-proline and sodium hydride were purchased from Sigma Aldrich. Dimethylcarbonate was purchased from Across Organics. Concentrated HCl and solvents were purchased from Fisher Scientific. All chemicals were used directly without purification.

Chromatographic purifications of products were performed on a flash-prep LC system puriFlash® 4100 from Interchim with prepacked silica column (30 µm, Interchim PF-Si30-HP), dual wavelength collection (λ = 254 and 320 nm) and a mixture of cyclohexane/ethyl acetate as eluant. ^{1}H NMR spectra were recorded on a Brucker Fourier 300 (300 MHz) and were calibrated with residual DMSO-d_6 protons signal at δ 2.50 ppm. Data are reported as follows: chemical shift (δ ppm), multiplicity (s = singlet, d = doublet and m = multiplet), integration, coupling constant (Hz) and assignment. ^{13}C NMR spectra were recorded on a Brucker Fourier 300 (75 MHz) and were calibrated with DMSO-d_6 signal at δ 39.52 ppm. Data are reported as follows: chemical shift (δ ppm) and attribution. All NMR assignments were made using COSY, HMBC and HSQC spectrum. Melting points were recorded on a Mettler Toledo MP50 Melting Point System ($T_{initial}$: 75 °C; Heating: 3 °C/min) with ME-18552 sample tubes. High resolution mass analyses (HRMS) were performed on an Agilent 1290 system, equipped with a PDA UV detector, and a 6545 Q-ToF mass spectrometer (Wilmington, DE, USA). The source was equipped with a JetStream ESI probe operating at atmospheric pressure.

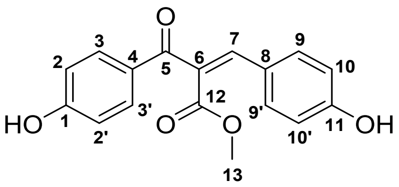

^{1}H NMR (300 MHz, DMSO-d_6): δ (ppm) 7.75 (m, 3H, H_3+$H_{3'}$+H_7), 7.22 (d, 2H, J = 8.7 Hz, H_9+$H_{9'}$), 6.83 (d, 2H, J = 8.7 Hz, H_2+$H_{2'}$), 6.67 (d, 2H, J = 8.7 Hz, H_{10}+$H_{10'}$), 3.66 (s, 3H, H_{13}). **^{13}C NMR** (75 MHz, DMSO-d_6): δ (ppm) 193.6 (C_5), 165.4 (C_{12}), 163.1 (C_1), 159.9 (C_{11}), 141.2 (C_7), 132.4 (C_9+$C_{9'}$), 131.6 (C_3+$C_{3'}$), 127.5 (C_4), 127.2 (C_6), 123.6 (C_8), 115.9 (C_{10}+$C_{10'}$), 115.8 (C_2+$C_{2'}$), 52.2 (C_{13}). **Mp** (°C): 192 – 194. **HRMS** (*m/z*) $[M-H]^-$ calcd for $C_{17}H_{14}O_5$: 297.0768; found: 297.0772.

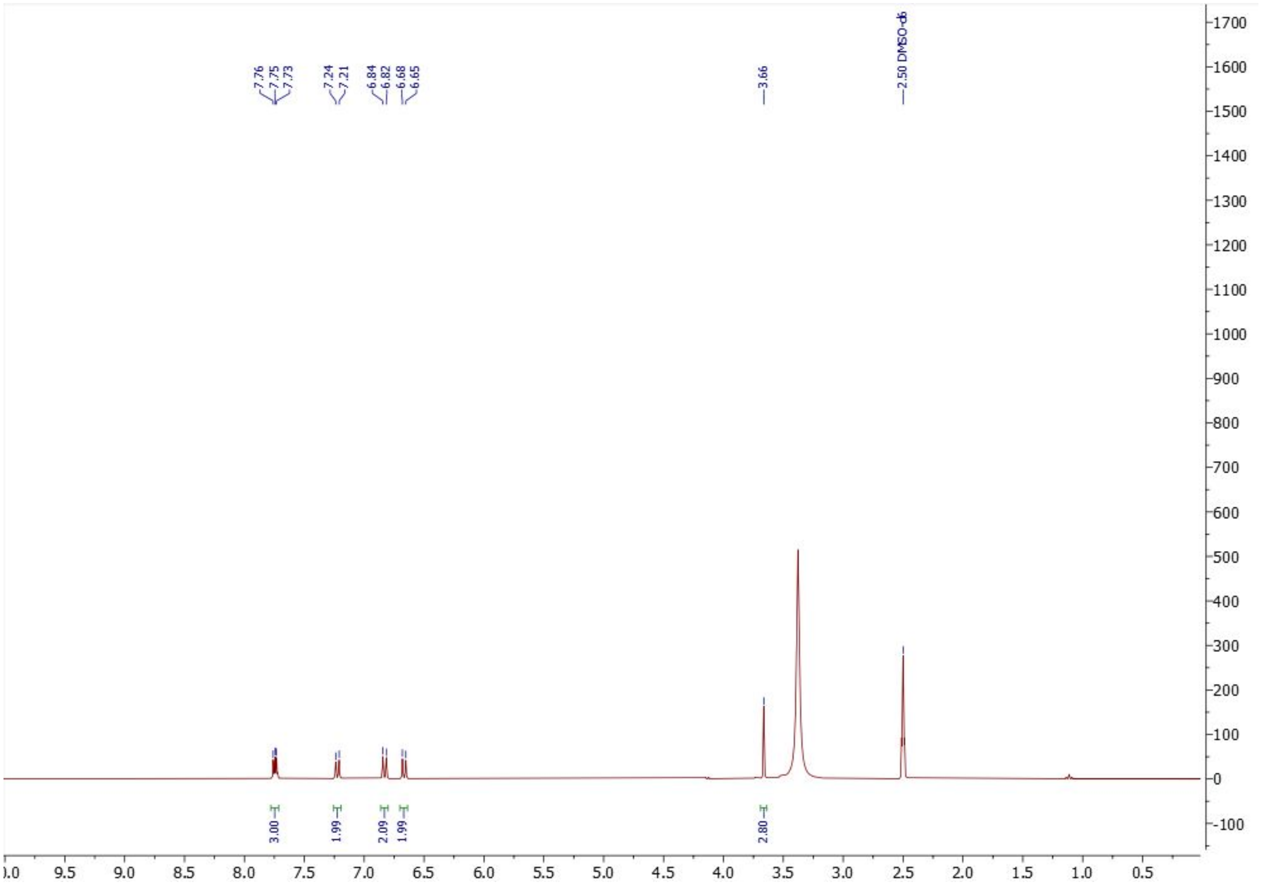

Figure S1. ^{1}H NMR spectra of substituted chalcone **B**

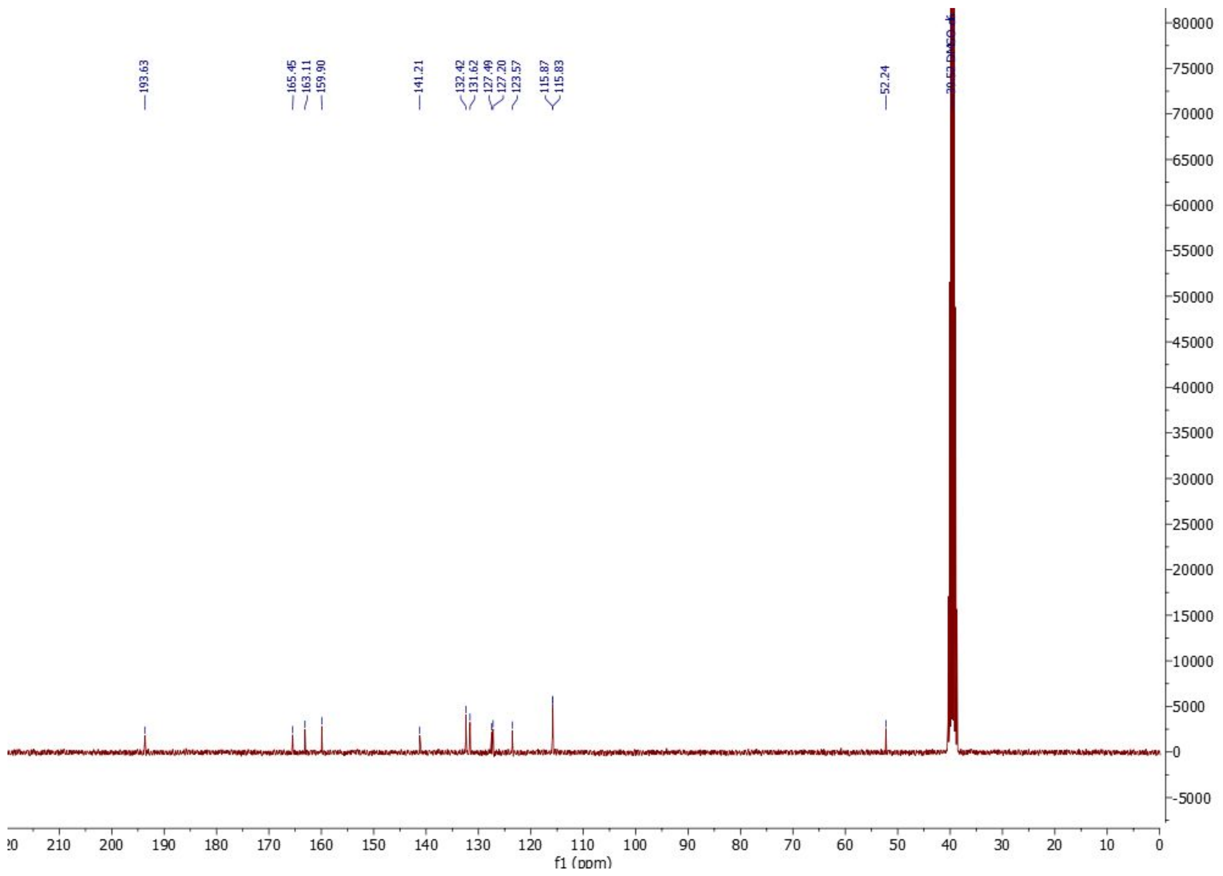

Figure S2. ^{13}C NMR Spectra of substituted chalcone **B**

2. Spectroscopy

Experimental and simulated UV-visible properties

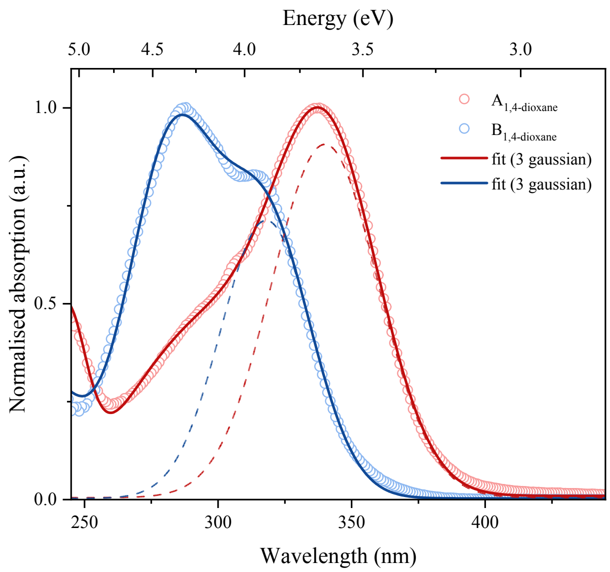

Figure S3. Gaussian functions simulating the absorption spectrum of **A** and **B** in 1,4-dioxane. Open circles: recorded absorption data. Overlaid: fit (solid) and longest wavelength Gaussian component (dashed).

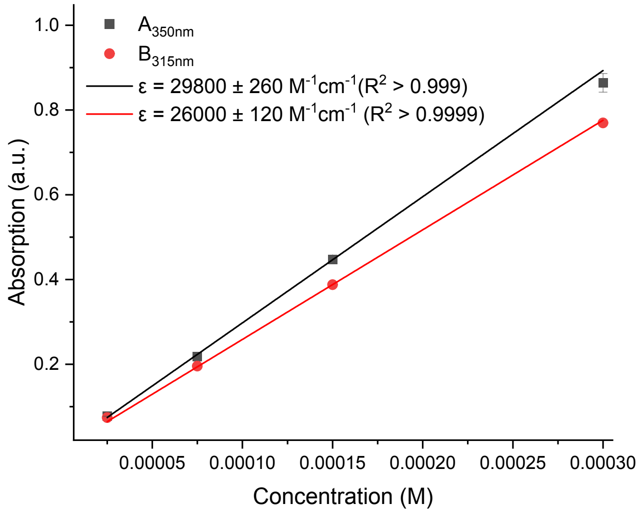

Figure S4. Absorption for **A** and **B** in ethanol at 350 nm and 315 nm, respectively (points), with calculated extinction coefficients fitted (lines).

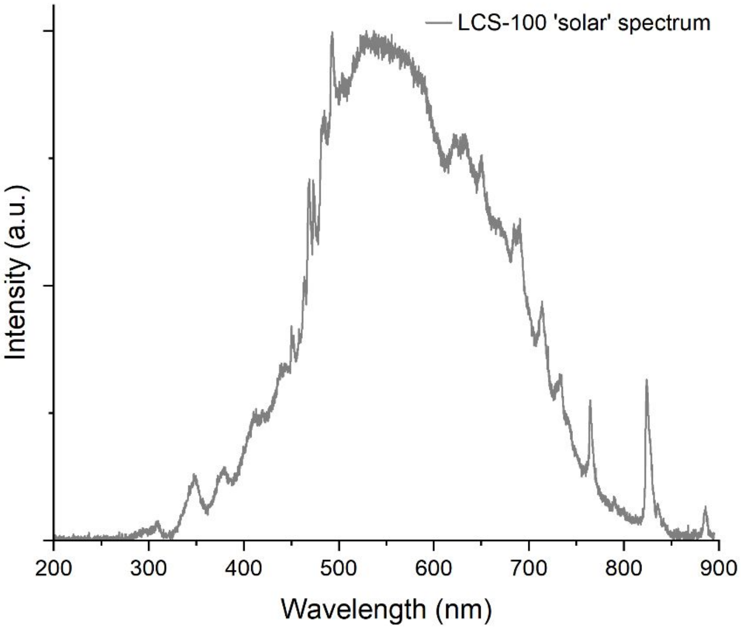

Figure S5. Output spectrum from the LCS-100 solar simulator. In solar photostability experiments, the sample was positioned such that irradiance was equivalent to one Sun on a clear day in summer on the surface of Earth (~1000 W/m^2).

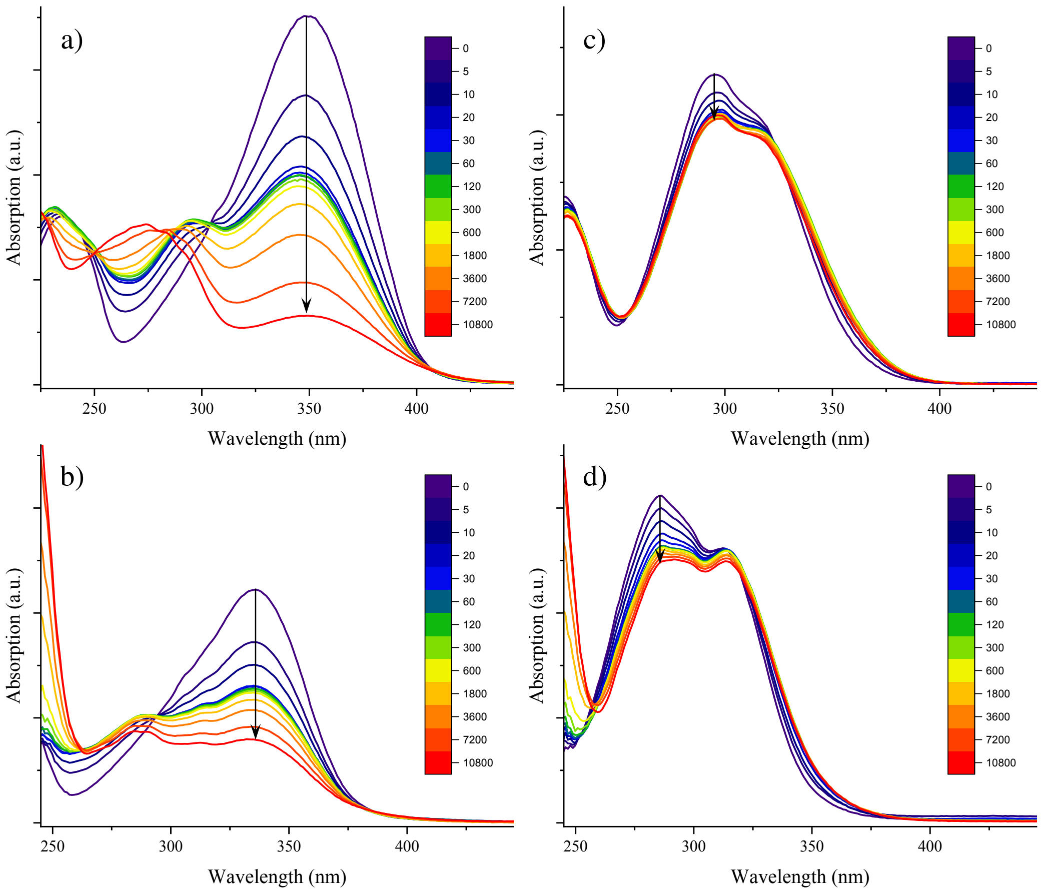

Figure S6. Absorption spectra for **A** and **B** in ethanol (a, c) and in 1,4-dioxane (b,d) throughout 3 h 'solar' irradiation (duration in s).

Area under curve index (AUCI) is defined as the ratio of integrated absorption spectra (280-400 nm) before (A_0) and after ($A_{2\,h}$) 2 h 'solar' irradiation:

$$AUCI(\%) = 100 \times \frac{\sum_{280}^{400} A_{2\,h}(\lambda)\Delta\lambda}{\sum_{280}^{400} A_0(\lambda)\Delta\lambda}$$

$$AUCI_{A-ethanol}(\%) = 100 \times \frac{25.87}{62.83} = 41\%$$

$$AUCI_{A-dioxane}(\%) = 100 \times \frac{18.71}{34.17} = 55\%$$

$$AUCI_{B-ethanol}(\%) = 100 \times \frac{31.59}{32.48} = 97\%$$

$$AUCI_{B-dioxane}(\%) = 100 \times \frac{37.74}{38.16} = 99\%$$

3. Computations:

Ground state geometries

As discussed in the main text, optimized geometries of the most stable identified structures of **A** and **B** (Figure 1) and the 9 next lowest lying conformers are presented in Table S1. The energies of each of these structures were very close. In **A**, the only structural differences arise from orientations of the OH groups at positions C_4 and C_{16}, and the position of the carbonyl group relative to the α, β-unsaturated double bond. **B** has more vibrational degrees of freedom and, due to its flexible nature, rotation around the substituent attached to C_{11} is possible, leading to more conformers in the ground state. These conformers were optimized at the DFT/B3LYP level, and then at the MP2/cc-pVDZ level to calculate more accurate energies.

According to the optimized geometry parameters of **A** and **B** (Figure 1), the optimal bond lengths of C_7 and O_{19} are very similar in both structures (1.23Å in **A** and 1.22Å in **B**). Significant differences are observed in the dihedral angles of the two structures. The aromatic rings in these structures have markedly different rotational barriers. The optimized structure of **A** is essentially planar. The dihedral angles of C_{13}-C_{12}-C_{11}-C_7 and H_{21}-C_{12}-C_{11}-H_{20}, have been determined to the 1° and 2°, respectively (see Figure 1, main text for numbering). **B** is similarly planar, but the methoxylate connected to C_{11} locates out of this plane. The dihedral angles of C_{13}-C_{12}-C_{11}-C_7 and H_{20}-C_{12}-C_{11}-C_{30} in **B** were determined as 3° and 8°, respectively.

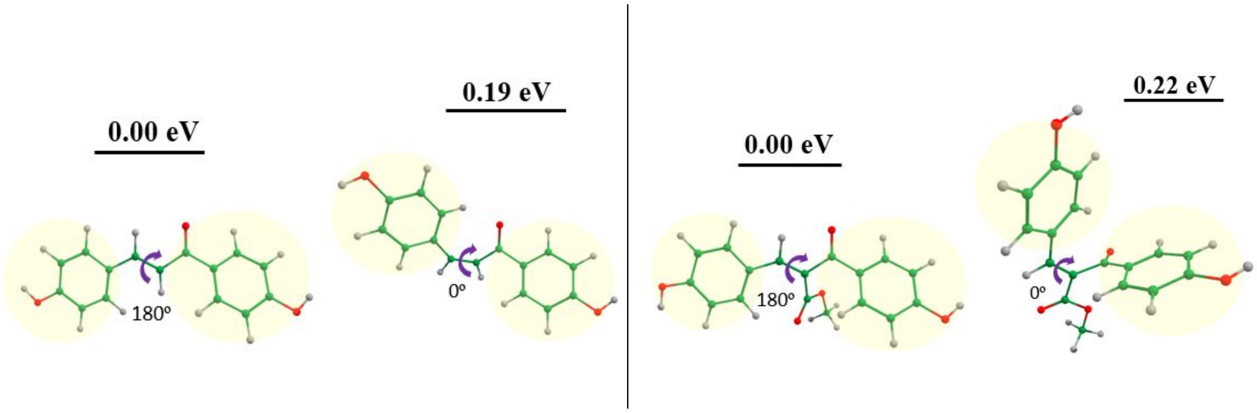

E-**A** *Z*-**A** *E*-**B** *Z*-**B**

Figure S7. Relative internal energy and stability for *Z* and *E* isomers of **A** and **B**, determined at the MP2/cc-pVDZ level.

Table S1. Optimized structures (along with relative energy in eV) for most stable identified conformers of **A** and **B**, determined at the MP2/cc-pVDZ level. Structures are ordered according to increasing energy. As shown, structure 1 has been assigned as the most stable (and is used in subsequent calculations).

**A Conformers**					
Label	1	2	3	4	5
Geometry	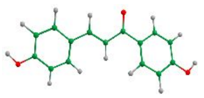	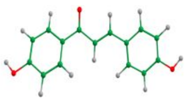	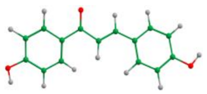	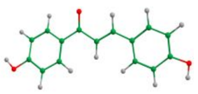	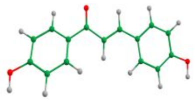
Relative Energy	0.00	0.01	0.01	0.01	0.01
Label	6	7	8	9	10
Geometry	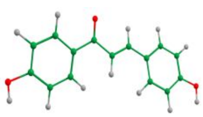	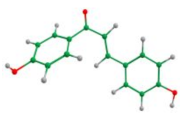	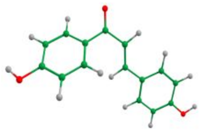	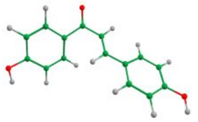	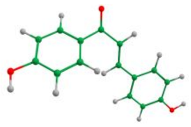
Relative Energy	0.01	0.05	0.05	0.06	0.06
**B Conformers**					

Label	1	2	3	4	5
Geometry	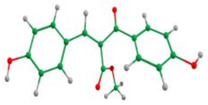	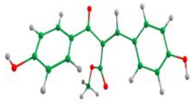	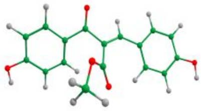	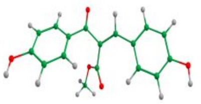	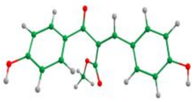
Relative Energy	0.00	0.01	0.01	0.01	0.01
Label	6	7	8	9	10
Geometry	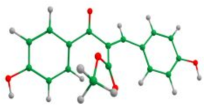	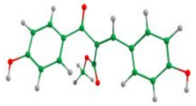	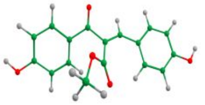	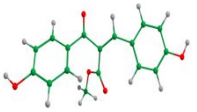	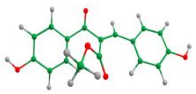
Relative Energy	0.02	0.02	0.03	0.05	0.05

Table S2. Contributed valence molecular orbitals in the S_1-S_4 electronic transitions for **A**, determined based on different theoretical levels.

**ADC(2)**					
State	Contributions	HOMO-5	HOMO	LUMO	LUMO+1
S_1 (nπ*)	L ← H-5 (80%)	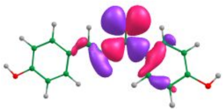	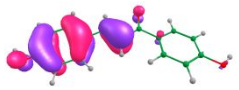	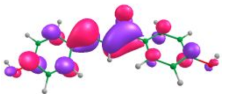	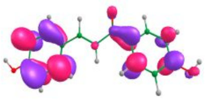
S_2 (ππ*)	L ← H (91%)				
S_3 (ππ*)	L+1 ← H (44%)				
S_4 (ππ*)	L ← H-1 (65%)				
**TD-DFT (PBE0)**					
		HOMO-2	HOMO-1	HOMO	LUMO
S_1 (nπ*)	L ← H-2 (69%)	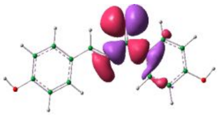	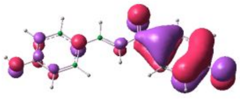	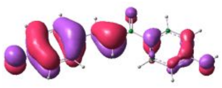	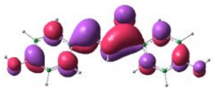
S_2 (ππ*)	L ← H (68%)				
S_3 (ππ*)	L ← H-1 (66%)				

<table>
<tr><td>S_4
($\pi\pi^*$)</td><td>L ← H-4
(48%)</td><td></td><td></td><td></td><td></td></tr>
<tr><td colspan="6">MS-CASPT2</td></tr>
<tr><td colspan="2"></td><td>HOMO-5</td><td>HOMO-1</td><td>HOMO</td><td>LUMO</td></tr>
<tr><td>S_1
($n\pi^*$)</td><td>L ← H-5
(81%)</td><td rowspan="4">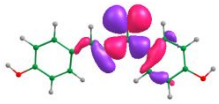</td><td rowspan="4">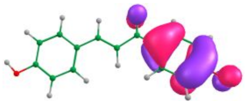</td><td rowspan="4">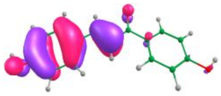</td><td rowspan="4">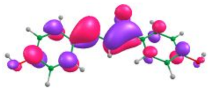</td></tr>
<tr><td>S_2
($\pi\pi^*$)</td><td>L ← H (83%)</td></tr>
<tr><td>S_3
($\pi\pi^*$)</td><td>L ← H-1
(46%)</td></tr>
<tr><td>S_4
($\pi\pi^*$)</td><td>L ← H-2
(43%)</td></tr>
</table>

Table S3. Contributed valence molecular orbitals in the S_1-S_4 electronic transitions for **B**, determined based on different theoretical levels.

**ADC(2)**					
State	Contributions	HOMO-5	HOMO	LUMO	LUMO+3
S_1 (nπ*)	L ← H-5 (47%)	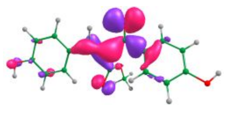	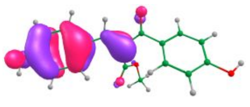	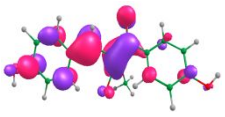	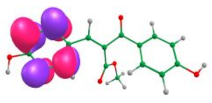
S_2 (ππ*)	L ← H (86%)				
S_3 (ππ*)	L+3 ← H (47%)				
S_4 (ππ*)	L ← H-1 (80%)				
**TD-DFT (ωB97XD)**					
		HOMO-2	HOMO-1	HOMO	LUMO
S_1 (nπ*)	L ← H-2 (56%)	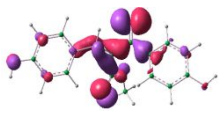	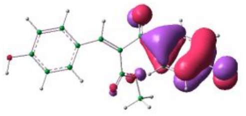	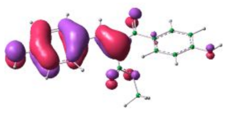	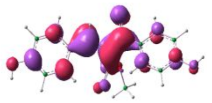
S_2 (ππ*)	L ← H (64%)				
S_3 (ππ*)	L ← H-1 (49%)				
S_4 (ππ*)	L ← H-3 (37%)				
**MS-CASPT2**					
		HOMO-5	HOMO-1	HOMO	LUMO
S_1 (nπ*)	L ← H-5 (53%)	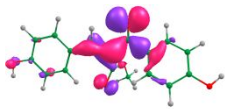	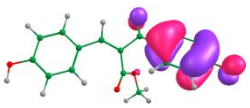	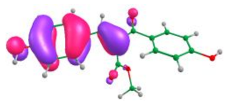	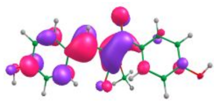
S_2 (ππ*)	L ← H (85%)				
S_3 (ππ*)	L ← H-1 (67%)				
S_4 (ππ*)	L← H-2 (72%)				

Dynamical properties:

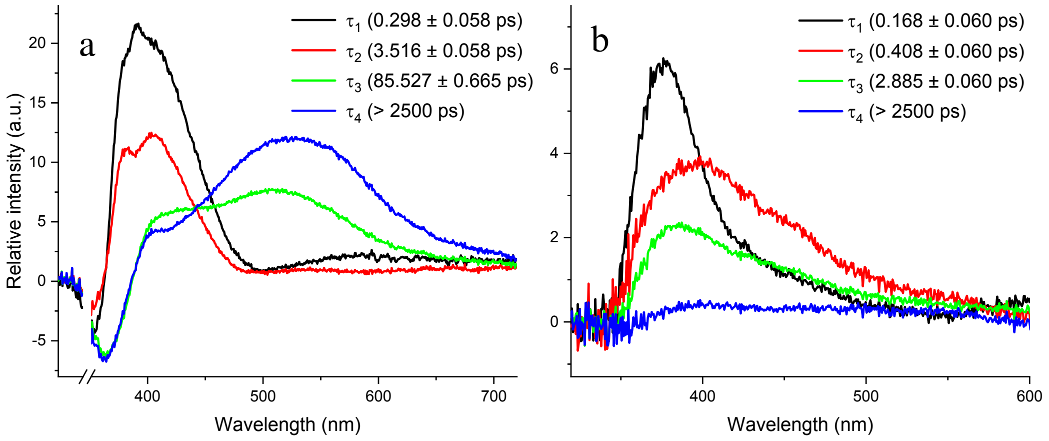

Figure S8. Evolution associated difference spectra (EADS) extracted from global sequential fitting of collected transient absorption data (Figure 4).

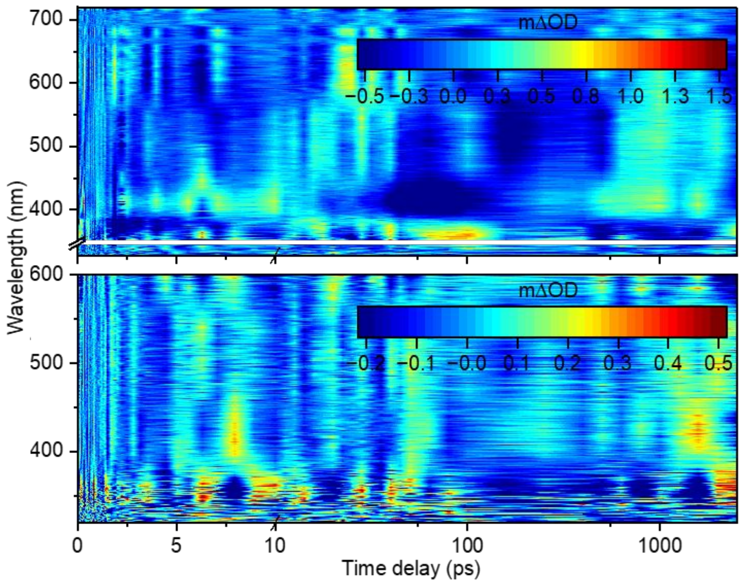

Figure S9. Residuals extracted from global sequential fitting of collected transient absorption data (Figure 4). Time scales are linear up to 10 ps and logarithmic thereafter.

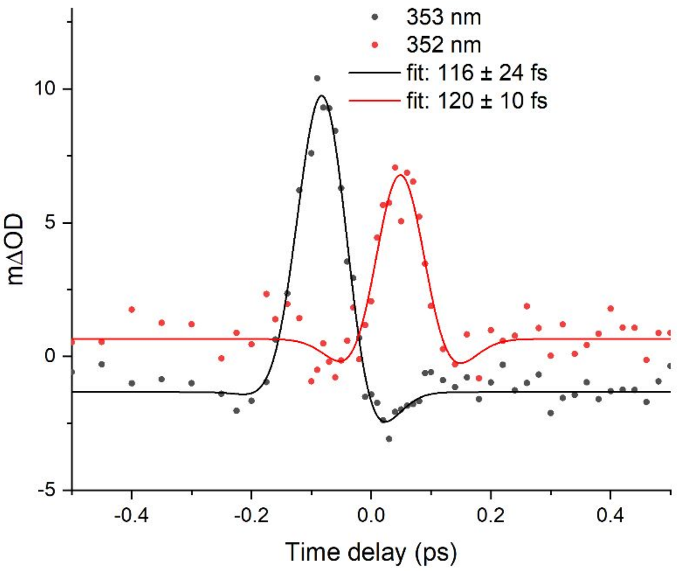

Figure S10. Kinetic transient absorption profiles of ethanol pumped at λ_{max} used in fs-TEAS experiments and probed at wavelengths near to this. Overlaid are fits, offset to account for non-baselined noise.

Kinetic transient absorption traces (Figure S10) were fitted with a Gaussian function and its first and second derivatives, according to:

$$y = e^{-\left(\frac{t-t_0}{FWHM/2\sqrt{\ln 2}}\right)^2} \cdot \left(A + B(t-t_0) + C(t-t_0)^2\right) \quad (S1)$$

where t_0 is time-zero and $FWHM$ is full width at half maximum of the convolution of two Gaussian pulses (i.e., 'pump' and 'probe'), to gauge instrument response function (IRF) for each experiment (see Table 2).

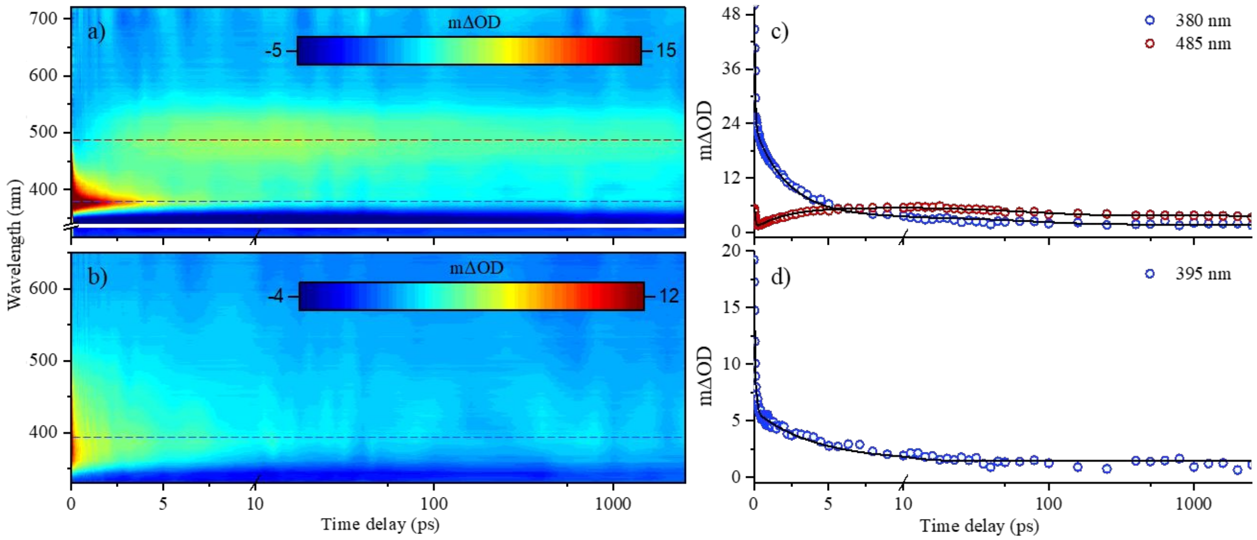

Figure S11. Collected transient absorption spectra and kinetic transient absorption traces at probe wavelengths labelled (with fits from global sequential analysis overlaid) of **A** (a, c) and **B** (b, d) in 1,4-dioxane, photoexcited at 335 and 320 nm respectively. Time scales are linear up to 10 ps and logarithmic thereafter.

Table S4. Lifetimes extracted from global sequential fitting of collected transient absorption data. Half of IRF (see Figure S10) is reported as the error in lifetime, except in cases where the error extracted from fitting is greater.

	$\mathbf{A}_{1,4\text{-dioxane}}$	$\mathbf{B}_{1,4\text{-dioxane}}$
τ_1 (ps)	0.069 < IRF	0.066 < IRF
τ_2 (ps)	2.263 ± 0.058	4.064 ± 0.060
τ_3 (ps)	77.428 ± 2.507	> 2500
τ_4 (ps)	> 2500	-

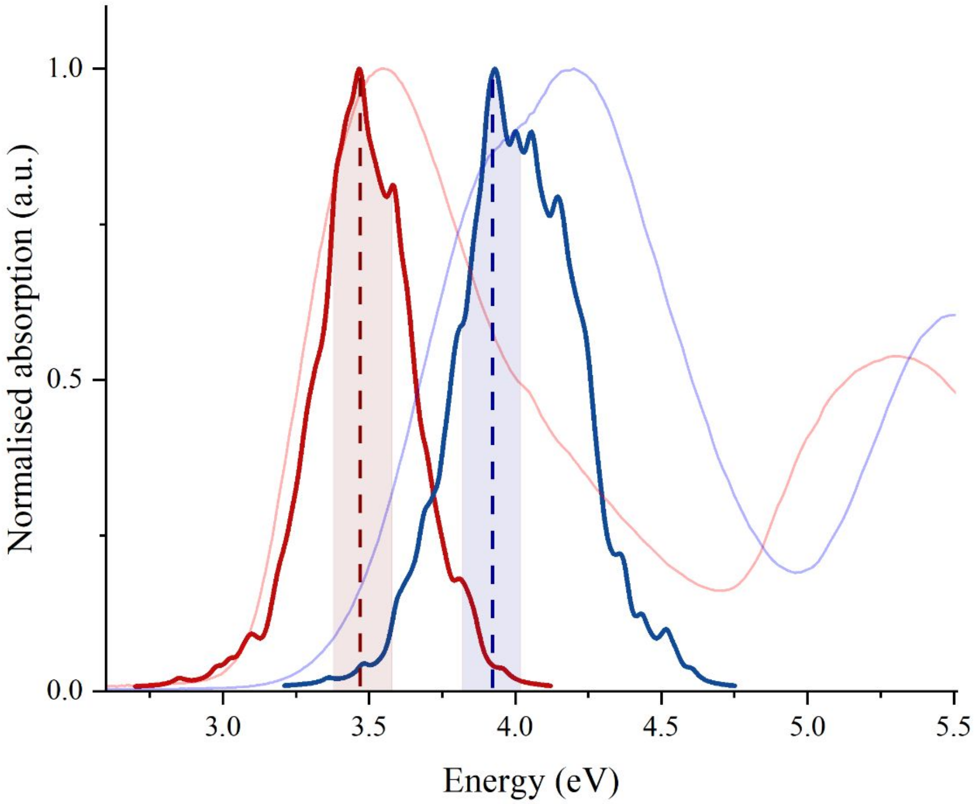

Figure S12. Normalized absorption spectra for **A** (solid dark red, simulated at the TD-PBE0/cc-pVDZ method, based on the $S_2 \leftarrow S_0$ electronic transition, in a PCM/ethanol implicit solvent with 500 points) and **B** (solid dark blue, simulated at the TD-ωB97XD/cc-pVDZ method, based on the $S_2 \leftarrow S_0$ electronic transition, in a PCM/ethanol implicit solvent with 500 points). The vertical dashed lines represent the absorption cross-section maxima (at 3.48 eV (356 nm) for **A** and at 3.92 eV (316 nm) for **B**); the shaded areas indicate the spectral window from which initial conditions for dynamics were sampled. Normalized absorption spectra of **A** (light red) and **B** (light blue) in ethanol are also presented.

Calculation of excited state lifetimes:

Following the nonadiabatic simulations, the total lifetimes for the S_2 and S_1 states in **B** have been determined based on the 94 trajectories initialized from S_2. Population traverses the S_2 PES towards the S_2/S_1 CI and finally decays to the ground state *via* another (S_1/S_0) CI. Curve fitting was undertaken based on the following phenomenological sigmoidal (logistic) equation[1, 2]:

$$y = \frac{a-b}{1+(t/\tau)^p} + b \qquad \text{(S2)}$$

where "τ" is the lifetime of the excited state.

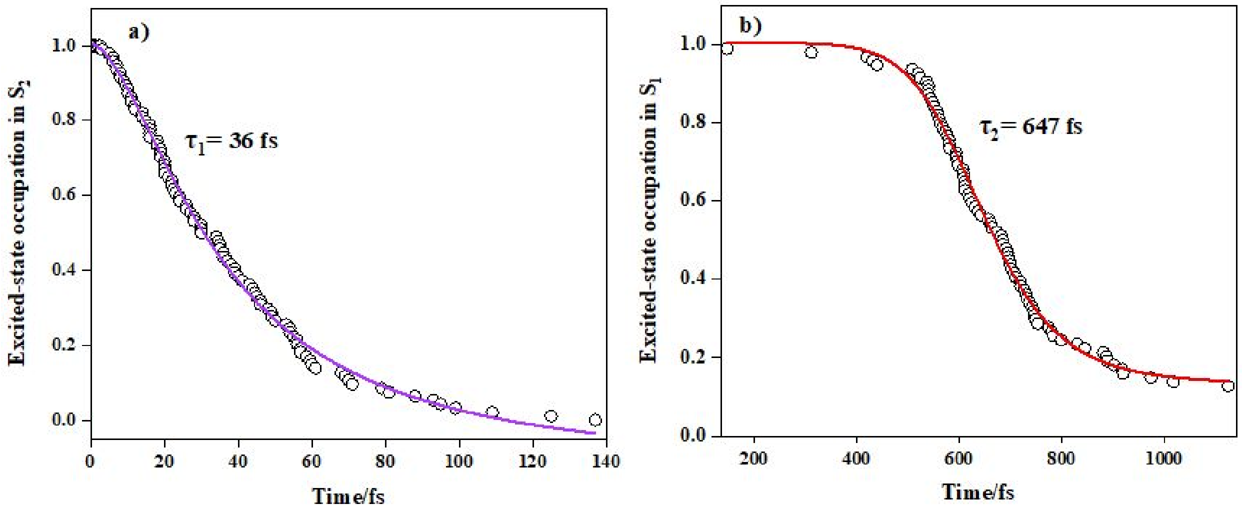

Figure S13. Approximate determination of the a) S_2 and b) S_1 excited state lifetime for chalcone **B** using a sigmoid equation fitting for state population versus time. The open circles represent the excited state population by simulation time, while the solid curve shows the fitted sigmoid function (see above), the extracted lifetimes from which are shown on each plot.

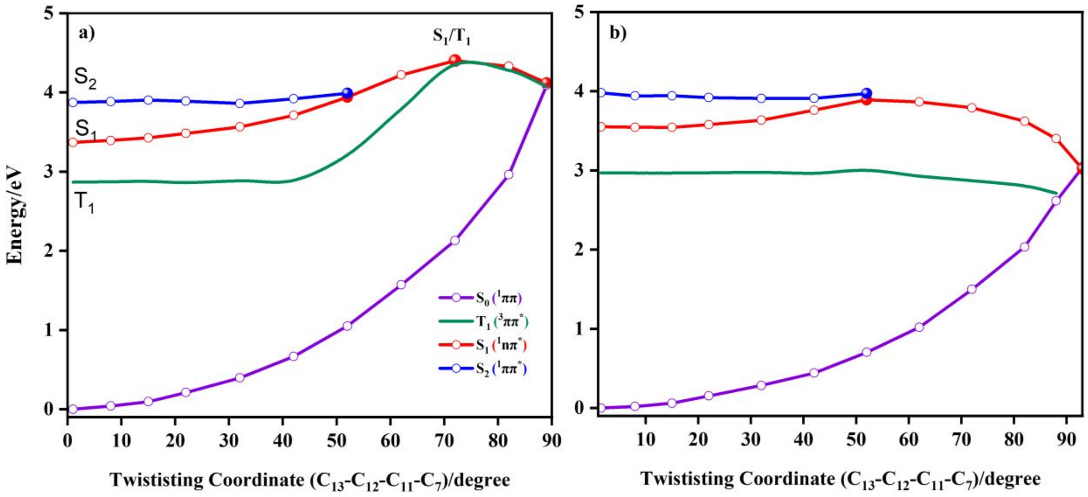

Figure S14. Potential energy profiles of the ground and excited states of **A** and **B**, including the first excited triplet state (T_1, green).

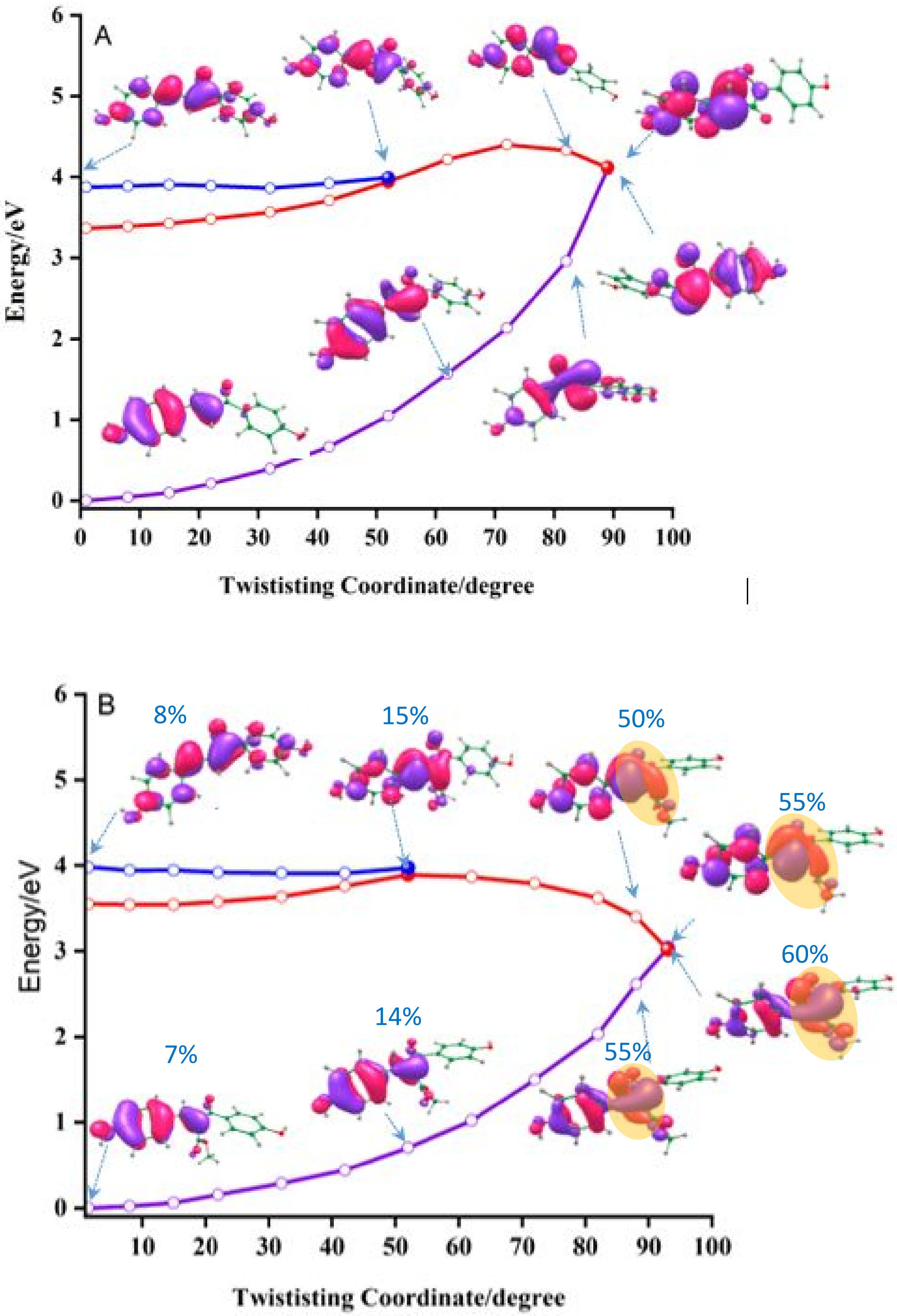

Figure S15: HOMO and LUMO orbitals of chalcone **A** (top) and **B** (bottom) corresponding to critical points in Figure 9 (manuscript). The relevant MOs corresponding to the optimized S_0, CI_1, CI_2 and an additional point before CI_2 have been depicted. To determine the contribution of α-methoxylate in the HOMO and LUMO of **B**, along with the twisting coordinate, natural bond orbital (NBO) analysis was performed using the Gaussian 16 and Chemissian programs. The contributions of α-methoxylate in each MO are presented (in %) in panel **B**.

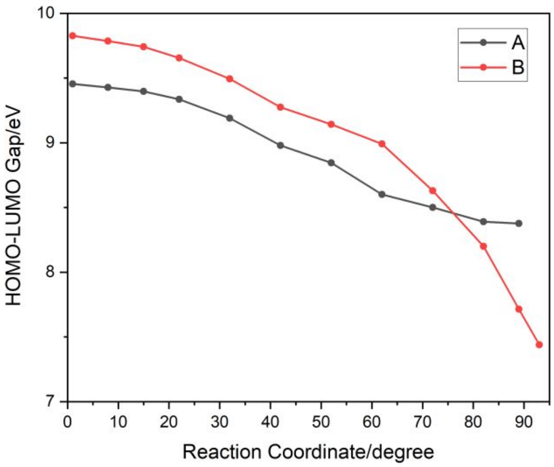

Figure S16. HOMO-LUMO energy gap for chalcone **A** and **B** along the twisting reaction coordinate corresponding. The HOMO-LUMO gap corresponds to the E_{LUMO}-E_{HOMO} (eV), and the reaction coordinate is rotation along the $C_{13}C_{12}C_{11}C_7$ dihedral, corresponding to the twisting coordinate in Figure 8 (manuscript) and Figure S13.

Cartesian coordinates from computation:

Table S4. XYZ coordinates of the ground state optimized geometry (S_0) of **A**, calculated at the MP2/cc-pVDZ level of theory.

30

C	2.61661	-0.93740	0.29918
C	2.49965	0.43119	-0.02942
C	3.86800	-1.56560	0.31591
C	5.02763	-0.83732	-0.00559
C	4.92162	0.52936	-0.33029
C	3.67061	1.15524	-0.33388
C	1.19419	1.17293	-0.04430
H	3.96914	-2.62265	0.57950
H	5.82343	1.10296	-0.57877
H	3.57683	2.21866	-0.57232
C	-0.06797	0.37708	-0.07251
C	-1.26500	1.00986	0.05005
C	-2.59865	0.40218	0.03051
C	-2.82013	-0.99009	-0.11086
C	-4.11330	-1.51794	-0.12587
C	-5.22998	-0.66738	0.00088
C	-3.72951	1.23955	0.15739
C	-5.03017	0.71708	0.14310
O	1.17812	2.40621	-0.04995
H	-0.01162	-0.70542	-0.21424
H	-1.22244	2.10094	0.17401
H	-1.97052	-1.67348	-0.20932
H	-4.28505	-2.59308	-0.23505
H	-3.58380	2.31996	0.26845
H	-5.89180	1.38872	0.24276
O	6.21723	-1.50832	0.01827
H	6.91203	-0.87554	-0.21670
H	1.73427	-1.52260	0.57392
O	-6.46565	-1.25013	-0.02165
H	-7.12176	-0.54403	0.07480

Table S5. XYZ coordinates of the optimised S_2/S_1 and S_1/S_0 conical intersections (CI_1-CI_2) geometries for **A**, calculated at the SA-CASSCF(6,6)/cc-pVDZ level of theory.

CI_1 (S_2/S_1)

30

C	2.59100	-0.92300	0.29800
C	2.49500	0.44800	-0.03200
C	3.83500	-1.56600	0.31900
C	5.00700	-0.85300	0.00100
C	4.92200	0.51600	-0.32700
C	3.67800	1.15600	-0.33500
C	1.19700	1.19600	-0.05000
H	3.91600	-2.62500	0.58400
H	5.83300	1.07300	-0.57500
H	3.60900	2.22000	-0.58300
C	-0.06600	0.40800	-0.07700
C	-1.26700	1.03900	0.04100
C	-2.42200	0.90700	-0.85200
C	-2.42800	0.07100	-1.99600
C	-3.55600	-0.01800	-2.81600
C	-4.71500	0.72700	-2.51500
C	-3.59400	1.64700	-0.56300
C	-4.72900	1.56100	-1.38100
O	1.18800	2.43300	-0.05900
H	-0.01100	-0.67600	-0.20500
H	-1.38800	1.72100	0.89500
H	-1.54000	-0.51900	-2.25500
H	-3.55800	-0.66600	-3.70100
H	-3.61000	2.30000	0.31700
H	-5.62600	2.14100	-1.14000
O	6.18500	-1.53700	0.02900
H	6.89800	-0.91900	-0.20700
H	1.70200	-1.50000	0.56600
O	-5.78100	0.59100	-3.35300
H	-6.49700	1.15700	-3.02100

CI_2 (S_1/S_0)

30

C	2.64500	-1.04000	-0.18000
C	2.40500	0.35500	-0.13300
C	3.91500	-1.54200	-0.13600
C	5.01500	-0.66600	-0.04400
C	4.80900	0.68800	-0.00400
C	3.49700	1.19800	-0.05300
C	1.03000	0.95200	-0.17000
H	4.10400	-2.60500	-0.17600
H	5.65100	1.36800	0.06200
H	3.32100	2.26200	-0.02800
C	-0.11600	0.18700	-0.07100
C	-1.19600	1.15300	0.04600
C	-1.95600	1.69300	-1.00600
C	-1.71500	1.31300	-2.35200
C	-2.44600	1.85200	-3.37400
C	-3.43800	2.80000	-3.08000
C	-2.95900	2.65600	-0.74400
C	-3.69700	3.20200	-1.75900
O	0.88200	2.41700	-0.31500
H	-0.21300	-0.78700	0.38400
H	-1.49500	1.52900	1.03000
H	-0.93600	0.58900	-2.53200
H	-2.28100	1.58100	-4.40500
H	-3.13900	2.96400	0.27800
H	-4.46300	3.94000	-1.55900
O	6.24200	-1.23500	-0.00400
H	6.90600	-0.56400	0.05200
H	1.81100	-1.72300	-0.26800
O	-4.11600	3.29300	-4.10000
H	-4.75600	3.93300	-3.82000

Table S6. XYZ coordinates of the ground state optimized geometry (S_0) of **B**, calculated at the MP2/cc-pVDZ level of theory.

36

C	3.59576	1.31210	-0.50562
C	2.41857	0.72552	-0.00252
C	4.85222	0.83228	-0.11743
C	4.95011	-0.22824	0.80452
C	3.77823	-0.80140	1.33288
C	2.52276	-0.33009	0.93006
C	1.10006	1.29886	-0.42161
H	5.76373	1.28648	-0.52591
H	3.87305	-1.60933	2.06472
H	1.62305	-0.77461	1.36767
C	-0.12387	0.41937	-0.27527
C	-1.30246	1.05575	-0.00850
C	-2.68204	0.58330	0.14489
C	-3.06523	-0.73317	0.49529
C	-4.41988	-1.06905	0.61431
C	-5.42498	-0.11039	0.38809
C	-3.70685	1.54547	-0.04877
C	-5.05829	1.20686	0.05694
O	1.01325	2.45459	-0.83116
H	-1.19427	2.14912	0.04187
H	-2.30145	-1.48480	0.69864
H	-4.69789	-2.09206	0.89801
H	-3.43072	2.57555	-0.30133
H	-5.84534	1.94958	-0.10445
O	6.14380	-0.74074	1.22741
H	6.84705	-0.23686	0.79160
H	3.50848	2.14641	-1.20804
O	-6.75928	-0.38713	0.48740
H	-6.84371	-1.32276	0.72384
C	0.05471	-1.03325	-0.57774
O	-0.36342	-1.99382	0.04857
O	0.81007	-1.14463	-1.69460
C	1.17107	-2.49780	-2.02319
H	1.75449	-2.94515	-1.20320
H	1.77822	-2.42409	-2.93390
H	0.27348	-3.10871	-2.20154

Table S7. XYZ coordinates of the optimised S_2/S_1 and S_1/S_0 conical intersections (CI_1-CI_2) geometries for **B**, calculated at the SA-CASSCF(6,6)/cc-pVDZ level of theory.

CI_1 (S_2/S_1)

36

C	3.60500	1.28600	-0.54300
C	2.41400	0.73400	-0.02900
C	4.85200	0.81900	-0.11000
C	4.92500	-0.19400	0.86700
C	3.73900	-0.73300	1.40500
C	2.49300	-0.27600	0.95600
C	1.10500	1.28000	-0.49400
H	5.77500	1.24100	-0.52500
H	3.81000	-1.50500	2.17700
H	1.58400	-0.69200	1.40200
C	-0.11500	0.40200	-0.32400
C	-1.29200	1.03600	-0.04100
C	-2.65100	0.92500	-0.57700
C	-3.16900	-0.17500	-1.30900
C	-4.48800	-0.16100	-1.77800
C	-5.32600	0.94600	-1.53700
C	-3.52000	2.01800	-0.32000
C	-4.83200	2.03800	-0.79800
O	1.01500	2.41400	-0.96700
H	-1.18500	1.82900	0.71400
H	-2.54800	-1.05500	-1.48400
H	-4.87900	-1.02400	-2.33200
H	-3.14700	2.87300	0.25800
H	-5.49100	2.89000	-0.60200
O	6.10500	-0.69000	1.33400
H	6.82700	-0.22400	0.88200
H	3.54600	2.07900	-1.29500
O	-6.61500	1.01000	-1.97500
H	-6.81100	0.19000	-2.46000
C	0.07700	-1.05300	-0.61400
O	-0.31700	-2.00800	0.03800
O	0.80400	-1.16800	-1.74300
C	1.17200	-2.52500	-2.08100
H	1.76700	-2.96700	-1.26800
H	1.76700	-2.44300	-2.99800
H	0.27300	-3.13500	-2.25400

CI_2 (S_1/S_0)

36

C	3.60500	1.28600	-0.54300
C	2.41400	0.73400	-0.02900
C	4.85200	0.81900	-0.11000
C	4.92500	-0.19400	0.86700
C	3.73900	-0.73300	1.40500
C	2.49300	-0.27600	0.95600
C	1.10500	1.28000	-0.49400
H	5.77500	1.24100	-0.52500
H	3.81000	-1.50500	2.17700
H	1.58400	-0.69200	1.40200
C	-0.11500	0.40200	-0.32400
C	-1.29200	1.03600	-0.04100
C	-2.38200	1.55400	-0.87000
C	-2.65600	1.18600	-2.21400
C	-3.72600	1.76000	-2.90700
C	-4.55100	2.71900	-2.29000
C	-3.24500	2.49900	-0.25600
C	-4.30700	3.08400	-0.95300
O	1.01500	2.41400	-0.96700
H	-1.42500	1.24300	1.03100
H	-2.04500	0.42500	-2.70200
H	-3.93100	1.45400	-3.94200
H	-3.06200	2.79000	0.78500
H	-4.96000	3.81900	-0.47200
O	6.10500	-0.69000	1.33400
H	6.82700	-0.22400	0.88200
H	3.54600	2.07900	-1.29500
O	-5.60400	3.30800	-2.92500
H	-5.65100	2.95500	-3.82900
C	0.07700	-1.05300	-0.61400
O	-0.31700	-2.00800	0.03800
O	0.80400	-1.16800	-1.74300
C	1.17200	-2.52500	-2.08100
H	1.76700	-2.96700	-1.26800
H	1.76700	-2.44300	-2.99800
H	0.27300	-3.13500	-2.25400

References

(1) Toldo, J. M.; Do Casal, M. T.; Barbatti, M. Mechanistic aspects of the photophysics of uva filters based on meldrum derivatives. *The Journal of Physical Chemistry A* **2021**, *125* (25), 5499-5508.

(2) Omidyan, R.; Shahrokh, L.; Whittock, A. L.; Stavros, V. G. Theoretical Insights into the Ultrafast Deactivation Mechanism and Photostability of a Natural Sunscreen System: Mycosporine Glycine. *The Journal of Physical Chemistry A* **2023**, *127* (22), 4880-4887.
